# Mitochondria Transfer from Mesenchymal Stem Cells Confers Chemoresistance to Glioblastoma Stem Cells through Metabolic Rewiring

**DOI:** 10.1158/2767-9764.CRC-23-0144

**Published:** 2023-06-14

**Authors:** Jean Nakhle, Khattar Khattar, Tülin Özkan, Adel Boughlita, Daouda Abba Moussa, Amélie Darlix, Frédérique Lorcy, Valérie Rigau, Luc Bauchet, Sabine Gerbal-Chaloin, Martine Daujat-Chavanieu, Floriant Bellvert, Laurent Turchi, Thierry Virolle, Jean-Philippe Hugnot, Nicolas Buisine, Mireille Galloni, Valérie Dardalhon, Anne-Marie Rodriguez, Marie-Luce Vignais

**Affiliations:** 1 Institute of Functional Genomics, University of Montpellier, CNRS, INSERM, Montpellier, France.; 2 Institute for Regenerative Medicine and Biotherapy, University of Montpellier, INSERM, CHU Montpellier, Montpellier, France.; 3 Institute of Molecular Genetics of Montpellier, University of Montpellier, CNRS, Montpellier, France.; 4 RESTORE Research Center, University of Toulouse, INSERM 1301, CNRS 5070, EFS, ENVT, Toulouse, France.; 5 Faculty of Medicine, Department of Medical Biology, University of Ankara, Ankara, Turkey.; 6 Department of Medical Oncology, Institut Régional du Cancer de Montpellier (ICM), University of Montpellier, Montpellier, France.; 7 Department of Pathology and Oncobiology, Hôpital Gui de Chauliac, Montpellier, France.; 8 The Center of the Biological Resource Center of University Hospital Center of Montpellier (BRC), Montpellier, France.; 9 Department of Neurosurgery, Hopital Gui de Chauliac, Montpellier, France.; 10 Toulouse Biotechnology Institute, University of Toulouse, CNRS, INRA, INSA, Toulouse, France.; 11 MetaboHUB-MetaToul, National Infrastructure of Metabolomics and Fluxomics, Toulouse, France.; 12 Université Côte D'Azur, CNRS, INSERM, Institut de Biologie Valrose, Team INSERM, “Cancer Stem Cell Plasticity and Functional Intra-tumor Heterogeneity”, Nice, France.; 13 UMR7221 Physiologie Moléculaire et Adaptation, CNRS, Muséum National d'Histoire Naturelle, Paris, France.; 14 Sorbonne Université, Institut de Biologie Paris-Seine (IBPS), CNRS UMR 8256, INSERM ERL U1164, Biological Adaptation and Ageing, Paris, France.

## Abstract

Glioblastomas (GBM) are heterogeneous tumors with high metabolic plasticity. Their poor prognosis is linked to the presence of glioblastoma stem cells (GSC), which support resistance to therapy, notably to temozolomide (TMZ). Mesenchymal stem cells (MSC) recruitment to GBM contributes to GSC chemoresistance, by mechanisms still poorly understood. Here, we provide evidence that MSCs transfer mitochondria to GSCs through tunneling nanotubes, which enhances GSCs resistance to TMZ. More precisely, our metabolomics analyses reveal that MSC mitochondria induce GSCs metabolic reprograming, with a nutrient shift from glucose to glutamine, a rewiring of the tricarboxylic acid cycle from glutaminolysis to reductive carboxylation and increase in orotate turnover as well as in pyrimidine and purine synthesis. Metabolomics analysis of GBM patient tissues at relapse after TMZ treatment documents increased concentrations of AMP, CMP, GMP, and UMP nucleotides and thus corroborate our *in vitro* analyses. Finally, we provide a mechanism whereby mitochondrial transfer from MSCs to GSCs contributes to GBM resistance to TMZ therapy, by demonstrating that inhibition of orotate production by Brequinar (BRQ) restores TMZ sensitivity in GSCs with acquired mitochondria. Altogether, these results identify a mechanism for GBM resistance to TMZ and reveal a metabolic dependency of chemoresistant GBM following the acquisition of exogenous mitochondria, which opens therapeutic perspectives based on synthetic lethality between TMZ and BRQ.

**Significance:**

Mitochondria acquired from MSCs enhance the chemoresistance of GBMs. The discovery that they also generate metabolic vulnerability in GSCs paves the way for novel therapeutic approaches.

## Introduction

Resistance to chemotherapy is a major obstacle for effective and long-lasting treatment of cancers, including glioblastomas (GBM). GBMs are aggressive brain tumors with poor prognosis. The current GBM standard of care consists of tumor resection followed by radiotherapy and chemotherapy with the DNA-alkylating agent temozolomide (TMZ; refs. 1–3). However, resistance to TMZ treatment develops quickly, mainly due to the presence of glioblastoma stem cells (GSC) within the tumor (4–7). GSCs can be cultured as neurospheres *in vitro* and their xenograft in mice leads to the formation of GBM tumors recapitulating the parental tumor heterogeneity. This heterogeneity is supported by the highly dynamic plasticity of GSCs, at both metabolic and transcriptional levels, which alters the mechanisms of drug responsiveness and the implementation of effective therapies (5, 6, 8–10).

The metabolic changes exhibited by tumors during cancer progression are known to contribute to resistance to treatment (11–14). Mitochondrial metabolism is central to these processes as it provides key metabolites for macromolecule synthesis, supporting cancer cell proliferation. Furthermore, increased production of metabolites linked to the mitochondrial tricarboxylic acid (TCA) cycle, such as succinate, fumarate, 2-hydroxyglutarate and α-ketoglutarate, also contribute to epigenetic deregulation of cancer cell gene expression by modifying the activities of DNA and histone demethylases (14–16). GSCs were reported to rely on diverse metabolic pathways including oxidative phosphorylation (OXPHOS), fatty acid oxidation and synthesis, as well as glutamine metabolism to sustain nucleotide biosynthesis (17–22).

Among biological means allowing cell interactions, physical connections through tunneling nanotubes (TNT) have recently emerged as major structures, which notably allow the cross-talk between tumors and their microenvironment (TME; refs. 23–27). TNTs are thin open-membrane tubular structures, which allow long-range intercellular connections and trafficking of various cargoes including mitochondria (28). TNT formation is enhanced by cellular stress, including reactive oxygen species (ROS) and ROS-inducing chemotherapies (26, 29, 30). TNTs and intercellular mitochondria transfers have been observed *ex vivo* in two-dimensional cell cultures and organoids and *in vivo* in human resected tumors and xenografts from a wide range of cancers (26, 29–34). In gliomas, TNT networks are generated between cancer cells and between cancer cells and cells of the TME. Both types of TNT networks have been shown to contribute to cancer cell plasticity and resistance to therapy (32, 35–37).

The recruitment of mesenchymal stem/stromal cells (MSC) to the GBM microenvironment was detected in resected tumors and in GSC orthotopic xenograft models where MSC tropism to the intracranial tumor is promoted by various factors including TGFβ, VEGF, MCP-1, and SDF-1 (38–41). The presence of MSCs in GBM is also increased following radiotherapy and inversely correlates with patient survival (41–43). MSCs participate to GBM progression by promoting GBM cell proliferation, invasiveness, angiogenesis, and resistance to therapy (42, 44–46).

MSCs were characterized for their capacity to establish intercellular TNT connections and to transfer mitochondria, to normal and cancer cells. MSC mitochondria induce metabolic and functional changes in the recipient cells, resulting in protection against tissue injury and, for cancer cells, in tumor progression and resistance to therapy, as shown by us and others (25, 33, 47–50). However, the cellular mechanisms by which MSC mitochondria trigger chemoresistance remain poorly documented.

In this study, we show that acquisition of exogenous MSC mitochondria by GSCs confers resistance to TMZ chemotherapy. We also show that MSC mitochondria induce a metabolic shift from glucose to glutamine utilization in GSCs, accompanied by a higher orotate turnover. Mechanistically, the TMZ resistance of GSCs depends on this higher orotate turnover, in relation to higher nucleotide biosynthesis. We also document increased nucleotide levels in resected GBM tumors from patients at relapse after TMZ treatment.

## Materials and Methods

### Cell Culture

The human primary GSCs used were previously published by the authors of the study [GB4 line (51), GB5 line (52)]. GB4 cells were isolated in J-P. Hugnot laboratory from a human GB resected at Gui de Chauliac Hospital in Montpellier and were characterized by comparative genomic hybridization (CGH) array. GB4 cells are from a male patient, they present an NF1 deletion as well as an EGFR, cMET, and BRAF amplification due to the presence of three chromosomes 7. GB5 cells were isolated in T. Virolle Laboratory (iBV Nice) from a human GB resected at Nice Hospital and characterized by CGH array. GB5 cells are also from a male patient, harbor three EGFR copies (linked to trisomy of chromosome 7), a homologous deletion of CDKN2A and a mutation of PTEN (R335 stop). GSCs were grown as neurospheres in DMEM: Nutrient Mixture F-12 (Gibco 21331046), supplemented with l-Glutamine (2 mmol/L; Gibco 25030024), D-Glucose (0.3%; Sigma-Aldrich G8769), bovine insulin (0.002%; Sigma-Aldrich I1882), N-2 supplement (Gibco 17502048), B-27 supplement (Gibco 12587010), EGF (10 ng/mL; Miltenyi Biotec 130-097-750), and FGF-2 (10 ng/mL; Miltenyi Biotec 130-104-924).

MSCs were isolated from the bone marrow of healthy donors at the authorized cell therapy unit (Biotherapy Team of General Clinic Research Center, French health minister agreement TCG/04/0/008/AA) at the Grenoble University Hospital. MSCs from 4 donors were used in this study and all experiments were performed with MSCs from at least 3 donors. MSCs were cultured in Minimum Essential Medium Eagle, alpha modification (Lonza Bioscience BE12-169F), supplemented with FBS (10%; Sigma-Aldrich F7524, lot no. BCBQ9326V), l-Glutamine (2 mmol/L), and FGF-2 (1 ng/mL). All cells were cultured at 37°C with 5% CO_2_ without antibiotics. Absence of *Mycoplasma* contamination was verified with MycoAlert *Mycoplasma* Detection Kit (Lonza LT07-118).

### Mitochondria Preparation

MSCs were trypsined without Ethylenediaminetetraacetic acid (EDTA) (Gibco 15090046) and then mechanically lysed in a buffer containing mannitol (200 mmol/L; Sigma-Aldrich M1902), saccharose (70 mmol/L; Sigma-Aldrich S0389), EDTA (1 mmol/L; Sigma-Aldrich E9884), HEPES (10 mmol/L, pH = 7.4; Sigma-Aldrich H3375) and 1X cocktail of protease and phosphatase inhibitors (Roche 04693159001), by using a syringe with 25- and 27-gauge needles. MSC mitochondria were isolated by two differential centrifugations at 800 × *g* and 8,000 × *g*, respectively. All steps of the mitochondria isolation were carried out at 0°C and the mitochondria pellet was finally resuspended in ice-cold mannitol buffer.

### Transfer of MSC Mitochondria to GSCs by Mitoception

The transfer of MSC mitochondria to GSCs was performed as described previously (49, 53, 54). Briefly, GSCs were seeded as a single-cell layer the day of the Mitoception. MSC mitochondria were isolated immediately prior to the Mitoception. They were serially diluted in ice-cold GSC culture medium and added to the GSCs which were then centrifuged at 3,000 rpm for 15 minutes. Mitocepted GSCs were incubated for 24 hours prior to collection for further analysis. Serial dilutions of MSC mitochondria were made considering the number of target GSCs. A ratio of 1 to 16 between the number of MSCs used for the mitochondria preparation and the number of target GSCs was found optimal to provide the observed biological effects. The actual rate of mitochondria transfer was determined by measuring the concentration of MSC mtDNA in the target GSCs.

### Mass Spectrometry Quantification

For cell metabolomics, cells were seeded on poly-lysine (Sigma-Aldrich P7280)-coated coverslips in complete DMEM-F12 medium. A control with poly-lysine but without cells was included. For tissues metabolomics, five 50-μm sections were rapidly made at −20°C for each human GBM tissue and the samples were returned to −80°C until processing for mass spectrometry. For all samples, the extraction was performed at −20°C in a solution of acetonitrile/methanol/water (2:2:1 v/v) containing formic acid (125 mmol/L) and isotope dilution mass spectrometry. After evaporation, samples were stored at −80°C until mass spectrometry analysis. Metabolite levels were normalized to cell numbers for cell samples and to protein concentrations for tissue samples. Mass spectrometry analysis details can be found in Supplementary Materials and Methods.

### ^13^C Stable Isotope Tracing Experiments

^13^C enrichment in intracellular metabolites was measured upon cell incubation with uniformly-labeled glucose (D-Glucose-^13^C_6_, Sigma-Aldrich 389374) or glutamine (l-Glutamine-^13^C_5_, Sigma-Aldrich 605166). Briefly, cells were seeded on poly-D-lysine–coated glass coverslips in complete DMEM-F12 medium (34 mmol/L glucose, 2 mmol/L glutamine). A total of 24 hours later, cells were thoroughly washed and placed in glucose-free and glutamine-free DMEM-F12 medium (BioWest L0091) supplemented with either 34 mmol/L labeled glucose and 2 mmol/L unlabeled glutamine or 2 mmol/L labeled glutamine and 34 mmol/L unlabeled glucose, for 24 and 48 hours. A control coverslip without cells was added for each experiment. Metabolites were extracted at −20°C in acetonitrile/methanol/water (2:2:1) containing formic acid (0.1%) and further processed (see Supplementary Materials and Methods).

### Patients Selection for GBM Tissue Analysis

Patients were retrospectively selected from the Biological Resources Center of the Montpellier University Hospital (BB-0033-00031) based on the following criteria: adult patient; at least two surgical resections for GBM (primary diagnosis and recurrence); tumor tissue from both surgeries available for analyses. Among the 8 selected patients, 5 were men. The median age at GBM diagnosis was 60.0 years (range, 19.1–66.3). Tumor was located in the right hemisphere for 5 patients, and in the left hemisphere for 3. Tumor location was frontal for 4 patients (50%), temporal for 2 patients (25%), and parietal and/or occipital for 2 patients (25%). Tumor classification as GBM was performed by an experienced neuropathologist (V. Rigau) by weighing together histopathologic information and molecular analyses, in accordance with the 2021 WHO classification of CNS tumors. Following the first surgery, all 8 patients received radiotherapy and chemotherapy with TMZ according to the Stupp protocol, with a median number of TMZ cycles of 7.5 (range, 3–13). Three patients received further oncological treatments due to tumor progression (various regimens of chemotherapy for 2 patients, second radiotherapy associated with TMZ for 1 patient). Second surgery for GBM was performed after a median of 15.7 months (range, 9.8–23.9) after the first surgery, of 13.6 months (range, 7.5–21.1) after the end of radiotherapy, and of 5.2 months (range, 2.0–10.1) after the end of adjuvant TMZ. All tumors were IDH1 wild-type. Median Ki67 values were 40% (range, 25%–60%) at first resection and 30% (range, 15%–50%) at second resection. All patients were deceased at the time of the study, with a median overall survival of 26.0 months (range, 14.8–38.4) after the first surgery and 11.3 months (range, 1.6–16.7) after the second surgery. Tissue samples from the 16 adult GBM tumors (8 patients) were collected at surgery, transported on ice to the pathology laboratory, frozen in liquid nitrogen, and stored at −80°C. Frozen GBM patient-derived tumors were obtained from the Center of Biological Resources of Montpellier Hospital and approved by the Institutional Review Board at Montpellier University Hospital (approval no.: IRB-MTP_2022_06_202201162).

### Statistical Analysis

Statistical analyses were carried out using GraphPad Prism 8 (GraphPad Software Inc., RRID:SCR_002798). Statistical assays were performed as described in each Figure legend. Multiple samples were analyzed by one-way ANOVA and Tukey *post hoc* test to evaluate statistical differences among the samples. Differences were considered statistically significant for *P* < 0.05 (*, *P* < 0.05; **, *P* < 0.01; ***, *P* < 0.001).

Schemes were made with BioRender.com (RRID:SCR_018361).

Additional information can be found in the Supplementary Materials and Methods section.

### Data Availability Statement

The data generated in this study are available upon request from the corresponding author.

## Results

### Human GSCs and MSCs Display Dynamic Interactions Resulting in the Acquisition of MSC Mitochondria by GSCs

**FIGURE 1:**
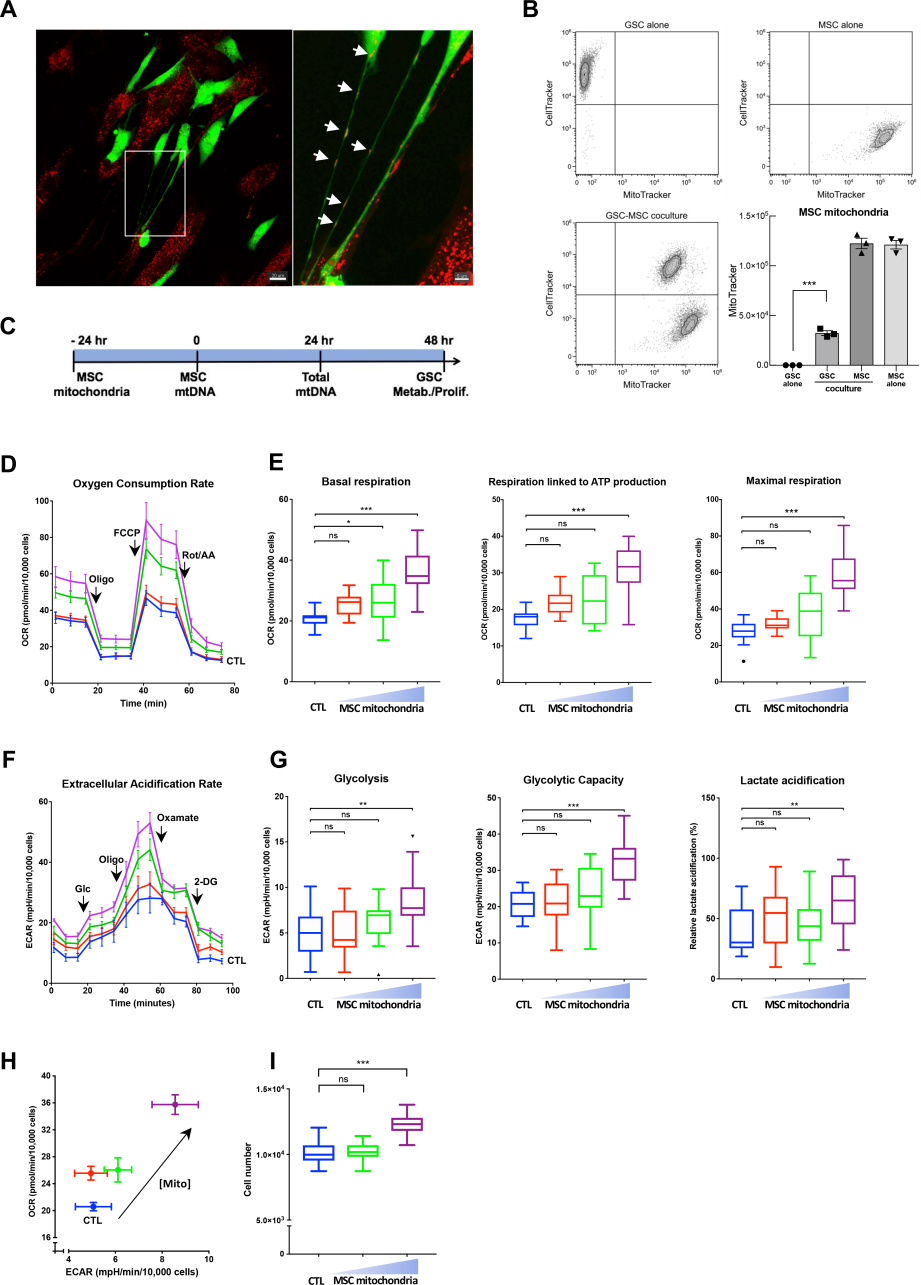
Exchange of mitochondria between MSCs with GSCs enhances GSC energy metabolism and proliferation. **A** and **B,** Mitochondria exchange during coculture of MSCs and GSCs, respectively prelabeled with red MitoTracker and green CellTracker. **A,** Imaging by confocal microscopy (24 hours). Scale bars: left, 20 μm; right, 5 μm. Arrows: MSC mitochondria. **B,** Quantification of mitochondria transfer to GSCs by flow cytometry analysis (48 hours coculture) Representative experiment and quantification with MSC from 3 donors. **C**–**I**, MSC mitochondria (three concentrations with 2-fold incremental increases) were transferred to GSCs by Mitoception and their effects on GSC functions were analyzed at 48 hours. **C,** Time line. **D–H,** Dose–response effects of MSC mitochondria on GSCs OCRs (D, E) and ECARs (F, G). All values were normalized to GSC cell numbers. **D,** Representative plot of GSC OCR in basal conditions and after sequential addition of oligomycin, Carbonyl cyanide-p-trifluoromethoxyphenylhydrazone (FCCP), and rotenone/antimycin. Mean values and SEM are indicated (*n* = 4). **E,** Tukey boxplots showing basal respiration, respiration linked to ATP production and maximal respiration. *n* = 18 from four independent experiments. One-way ANOVA; *, *P* < 0.05; ***, *P* < 0.001. **F,** Representative plot of GSC ECAR in basal conditions and after sequential addition of glucose, oligomycin, oxamate, and 2-deoxyglucose. Mean values and SEM are indicated (*n* = 6). **G,** Tukey boxplots showing basal glycolysis, glycolytic capacity, and lactate acidification. *n* = 13 from three independent experiments. One-way ANOVA; *, *P* < 0.05; **, *P* < 0.01; ***, *P* < 0.001. **H,** OCR versus ECAR of GSCs with MSC mitochondria. Mean and SEM. **I,** Tukey boxplots showing GSCs proliferation. One-way ANOVA; ***, *P* < 0.001. Data from B to I were obtained with MSCs from 3 donors.

To test whether GSCs and MSCs can establish physical connections and exchange mitochondria, we set up cocultures of red MitoTracker-labeled MSCs and green CellTracker-labeled GSCs, which were analyzed by time-lapse imaging and confocal microscopy (Supplementary Video S1; Fig. 1A). MSCs and GSCs demonstrated dynamic interactions, through TNT-like protrusions, some of which were maintained for up to 14 hours and led to the transfer of MSC mitochondria to the GSCs (Supplementary Video S1). At 24 hours of coculture, MSC mitochondria were visualized both inside the TNTs connecting MSCs to GSCs and in the TNT-connected GSCs (Fig. 1A). The extent of MSC mitochondria acquisition by GSCs following cocultures was further quantified by monitoring the MSC mitochondria fluorescence in GSCs by flow cytometry (Fig. 1B).

### MSC Mitochondria Enhance GSC Energy Metabolism and Proliferation

To characterize the effects of MSC mitochondria acquired by GSCs, we used our previously described Mitoception protocol which allows mitochondria dose–response analysis (49, 54). Briefly, this approach is based on the quantitative transfer of preisolated MSC mitochondria to GSCs. The day following Mitoception, considered as time zero (Fig. 1C; Supplementary Fig. S1), the amount of transferred exogenous MSC mitochondria can be quantified, relative to the endogenous GSC mitochondria, based on the concentrations of their respective mitochondrial DNAs (mtDNA; ref. 53).

We first evaluated the effects of MSC mitochondria on GSC energy metabolism, by using the Seahorse technology, 48 hours after the transfer, by Mitoception, of 2-fold increasing concentrations of MSC mitochondria. We found that MSC mitochondria increased the oxygen consumption rate (OCR) of the recipient GSCs in a dose-dependent fashion, as compared with control GSCs (Fig. 1D and E). This increase in OXPHOS was observed for basal respiration, respiration linked to ATP production and maximal respiration, with respectively 1.7-, 1.8-, and 2.1-fold increases for the most effective MSC mitochondria concentration.

Likewise, measurements of the extracellular acidification rate (ECAR) demonstrated increases in basal glycolysis, maximal glycolytic capacity, and acidification linked to lactate production, of respectively 1.7-, 1.6-, and 2.3-fold (Fig. 1F and G). As shown by the energy—OCR versus ECAR—plot, GSCs underwent concomitant increases in OXPHOS and glycolysis following increasing amounts of MSC mitochondria acquisition, further indicating that MSC mitochondria not only enhanced OXPHOS, which is directly dependent on mitochondrial activity, but more generally the overall GSC energy metabolism (Fig. 1H). MSC mitochondria also enhanced GSC proliferation, as shown by the 1.2-fold increase in GSC cell number at 48 hours for the higher MSC mitochondria concentration (Fig. 1I).

We quantified the amounts of transferred MSC mitochondria generating these effects in GSCs on the basis of mtDNA concentrations. Because MSCs and GSCs originate from different donors, their mtDNAs could be distinguished on the basis of SNPs. The maximal metabolic effects were obtained with small amounts of transferred MSC mitochondria, estimated to 0.4% of the GSC endogenous mitochondria content (Supplementary Fig. S2). As previously shown in other cell systems (49, 55), we found that acquisition of MSC mitochondria led to an increase in total mtDNA levels, 2.0-fold at 24 hours (Supplementary Fig. S2), suggesting that mitochondrial biogenesis was stimulated in GSCs. Overall, our results showed that acquisition of small amounts of MSC mitochondria triggers cellular processes in GSCs, including energy metabolism and proliferation.

### MSC Mitochondria Increase GSC Survival in Response to TMZ Treatment

**FIGURE 2:**
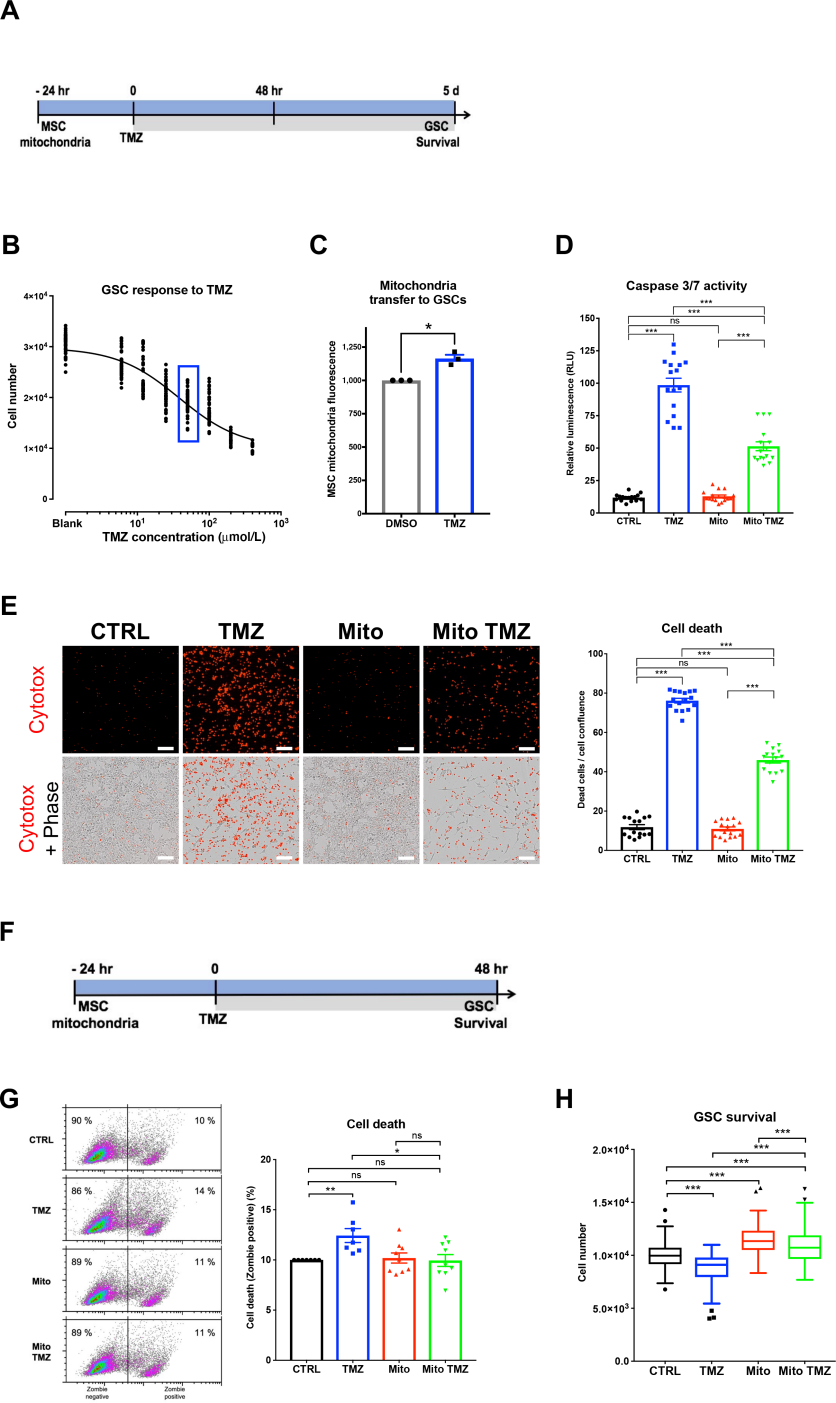
MSC mitochondria increase GSC survival in response to TMZ. **A** and **F,** Time lines for the response of GSCs to TMZ, at day 5 (**B–E**) and at 48 hours (**G–H**). **B,** Survival of GSCs in response to TMZ (dose–response 6–400 μmol/L). Framed TMZ concentration of 50 μmol/L used in all subsequent experiments. **C,** Effect of TMZ on the transfer of mitochondria from MSCs to GSCs, as analyzed by flow cytometry. **D** and **E,** Effects of MSC mitochondria on GSC survival in response to TMZ. **D,** GSC caspase 3/7 activity (*n* = 16, three independent experiments). **E,** Cytotox assay (Incucyte). Representative images and quantification of Cytotox labeled GSCs (*n* = 15, 3 independent experiments). **G** and **H,** Effects of MSC mitochondria on GSC survival in response to TMZ (48 hours). **H,** GSC cell death. FACS analysis of Zombie violet-stained GSCs. Representative data and quantification from seven independent experiments with mean and SEM values. **H,** GSC cell number (*n* = 84 from seven independent experiments). Tukey boxplots with Kruskal–Wallis test; **, *P* < 0.01; ***, *P* < 0.001. C–E and G, Statistical analysis by one-way ANOVA; *, *P* < 0.05; ***, *P* < 0.001.

We next investigated the effect of MSC mitochondria on GSC response to TMZ, as the metabolic activity of cancer cells is known to affect their response to therapy. We first performed TMZ dose–response experiments (range, 6–400 μmol/L) to assess viability of GSCs at day 5 of TMZ treatment (Fig. 2A and B). We determined a TMZ IC_50_ of 38 μmol/L (95% confidence interval: 33–44 μmol/L), consistent with the TMZ concentrations of 15–35 μmol/L reported in glioma tumors following TMZ administration (56). Accordingly, a TMZ concentration of 50 μmol/L was used in all subsequent experiments. Of note, incubation of MSCs and GSCs with this concentration of TMZ was found to enhance mitochondrial transfer from MSCs to GSCs, in 3-day cocultures (Fig. 2C).

To determine the effects of MSC mitochondria on the survival of GSCs to TMZ, the concentration of MSC mitochondria established above as the most effective in stimulating GSC energy metabolism and proliferation was used. At day 5 of TMZ treatment (50 μmol/L), TMZ increased GSC caspases 3/7 activities 8.3-fold and this effect was reduced 2-fold in GSCs with acquired MSC mitochondria (Fig. 2D). Likewise, the rate of TMZ-induced cell death, as measured by Cytotox staining, was reduced from 6.4-fold for control GSCs to 4.2-fold for GSCs with previously acquired MSC mitochondria (Fig. 2E). These results demonstrated that MSC mitochondria reduce TMZ-induced GSC cell death. As our goal was to identify early cellular mechanisms responsible for this GSC acquired resistance to TMZ, we checked whether MSC mitochondria affected GSC survival, as soon as 48 hours following TMZ treatment (Fig. 2F). At this early timepoint, MSC mitochondria were found to inhibit the small but already significant cell death induced by TMZ in GSCs (Fig. 2G and H). These data showed that MSC mitochondria acquisition by GSCs induces resistance to TMZ, with early signs as soon as 48 hours following TMZ treatment.

### MSC Mitochondria Alter the Metabolic Response of GSCs to TMZ

**FIGURE 3:**
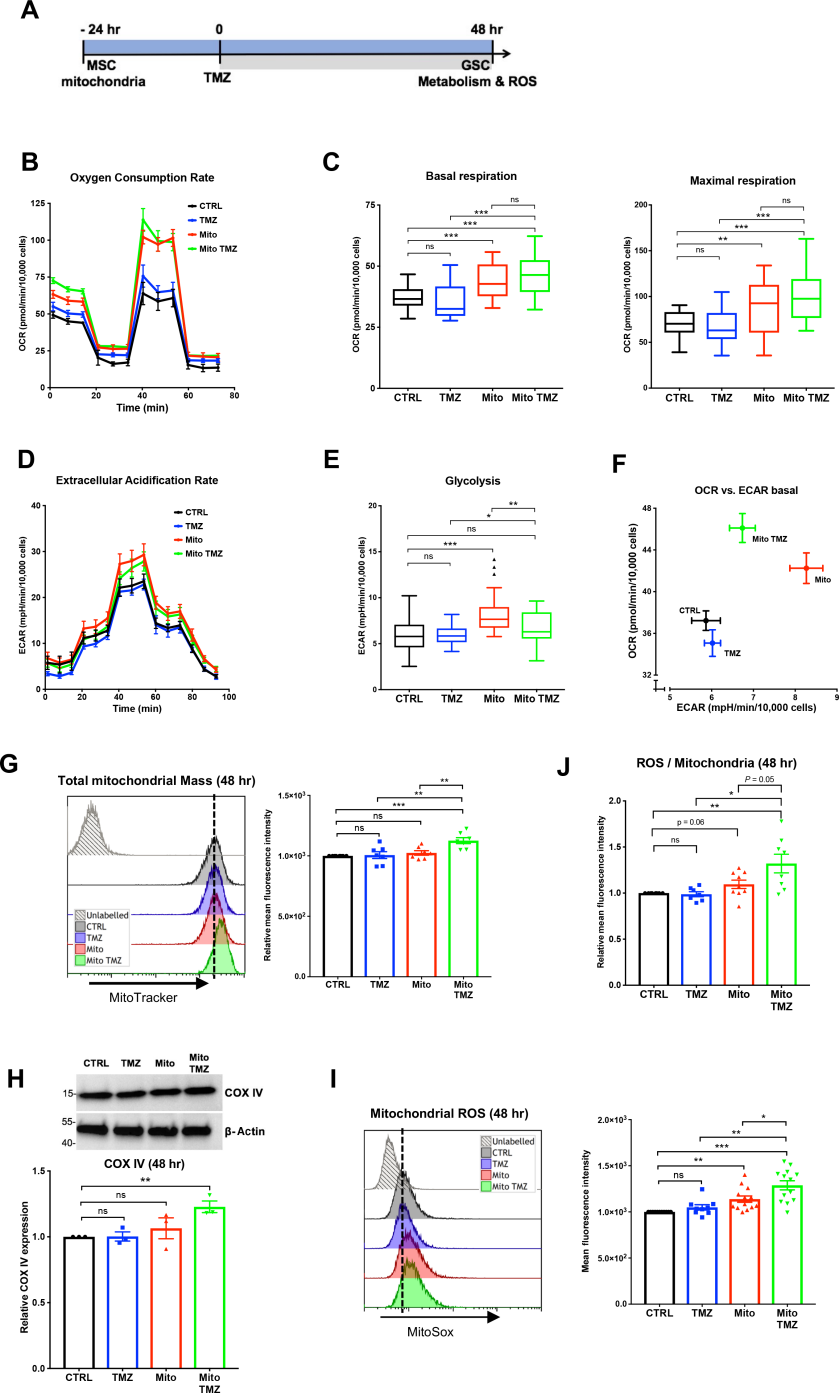
MSC mitochondria modify the metabolic response of GSCs to TMZ. MSC mitochondria were transferred by Mitoception to GSCs which were subsequently treated with TMZ (50 μmol/L) for 48 hours. **A,** Time line. Effects of TMZ in the presence/absence of MSC mitochondria on GSC OCRs (**B** and **C**) and ECARs (**D** and **E**). All values were normalized to GSC cell numbers. **B,** Representative plot of GSC OCR in basal conditions, treated with TMZ, MSC mitochondria or both, and after sequential addition of oligomycin, FCCP and rotenone/antimycin. Mean values and SEM (*n* = 4). **C,** Tukey boxplots showing basal respiration and maximal respiration (*n* = 25 from four independent experiments). One-way ANOVA; **, *P* < 0.01; ***, *P* < 0.001. **D,** Representative plot of GSC ECAR in basal conditions, treated with TMZ, MSC mitochondria or both, and after sequential addition of glucose, oligomycin, oxamate, and 2-deoxyglucose. Mean values and SEM (*n* = 5). **E,** Tukey boxplots showing GSC basal glycolysis (*n* = 25 from four independent experiments). One-way ANOVA; **, *P* < 0.01; ***, *P* < 0.001. **F,** OCR versus ECAR values of GSCs treated with TMZ, with MSC mitochondria or with both. Mean and SEM values. **G–J,** GSC mitochondrial mass and ROS production. GSCs labeled with MitoTracker and MitoSox were analyzed by FACS, following the acquisition of MSC mitochondria and 48 hours TMZ treatment. **G,** GSC total mitochondrial mass. Representative experiment and relative mitochondria mean fluorescence intensity values represented as mean ± SEM (*n* = 7). **H,** Expression of COX IV protein. Representative Western blots for COX IV and β−actin expression (MW markers in kDa). Quantifications (*n* = 3) represented as mean ± SEM. **I,** GSC ROS production as measured with Mitosox. Representative data and quantification from independent experiments (*n* = 9) with mean and SEM values. **J,** Ratios of GSC ROS production over mitochondrial mass (*n* = 7). G–J, One-way ANOVA; *, *P* < 0.05; **, *P* < 0.01; ***, *P* < 0.001.

We investigated whether MSC mitochondria altered the metabolic response of GSCs to TMZ (time scale; Fig. 3A). Whereas TMZ alone had little effect on GSC OXPHOS activity, it further enhanced the stimulatory effects of MSC mitochondria on GSC OXPHOS (Fig. 3B and C). TMZ alone did not affect GSC glycolysis either. However, it abrogated the increased glycolysis induced by MSC mitochondria (Fig. 3D and E). Overall, these data showed that the acquisition of MSC mitochondria by GSCs disrupts their metabolic response to TMZ, favoring OXPHOS over glycolysis (Fig. 3F).

We next tested whether this enhanced OXPHOS activity was associated with increased mitochondrial mass. Flow cytometry analysis of MitoTracker-labeled GSCs showed that cotreatment with MSC mitochondria and TMZ increased the total mitochondrial mass of GSCs, as observed at both 24 and 48 hours (Fig. 3G; Supplementary Fig. S3). Consistent with these observations, we also detected increased concentrations of cytochrome c oxidase IV (COX IV), a protein of the inner mitochondrial membrane (Fig. 3H; Supplementary Fig. S3). We assessed by MitoSOX staining whether this increase in GSC mitochondrial mass was associated with increased mitochondrial ROS production. While TMZ alone did not alter ROS production by GSCs, cotreatment with MSC mitochondria and TMZ triggered the production of ROS, as observed at 48 hours (1.3-fold; Fig. 3I) with a 1.3-fold increase in ROS/mitochondrial mass ratio compared with control GSCs (Fig. 3J). Overall, our data showed additive effects of MSC mitochondria and TMZ treatment on GSC mitochondrial activity, with increased OXPHOS, mitochondrial mass, and ROS production.

### MSC Mitochondria Alter Metabolite Production by GSCs in Response to TMZ

**FIGURE 4:**
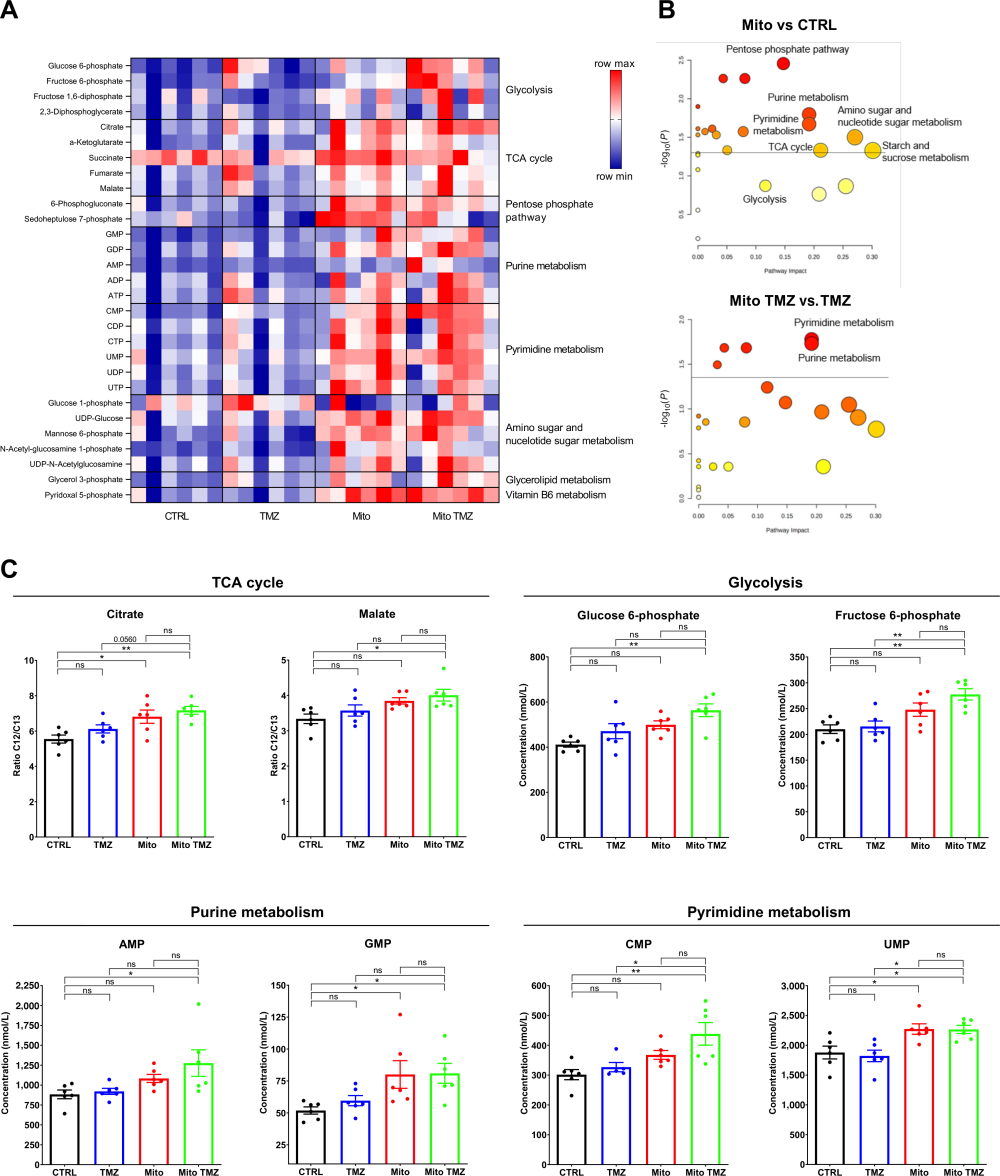
MSC mitochondria modify GSC metabolite production in response to TMZ. GSC metabolites production was analyzed by mass spectrometry on whole-cell extracts. **A,** Heat map of metabolites produced by GSCs following acquisition of MSC mitochondria and/or TMZ treatment. **B,** MSEA in GSCs after mitochondria acquisition in comparison with control GSCs, without or with TMZ treatment. **C,** Metabolite concentrations for metabolic pathways identified in B. Two independent experiments were performed, each in triplicate. Each point corresponds to an individual culture and extraction. Values were normalized to cell numbers. Means ± SEM and *t* tests with Welch correction; *, *P* < 0.05; **, *P* < 0.01.

To determine the impact on GSC metabolism of the cotreatment with MSC mitochondria and TMZ, we performed GSC metabolomics analysis. Acquisition of MSC mitochondria increased the levels of metabolites involved in diverse metabolic pathways, including the TCA cycle, purine and pyrimidine metabolism, amino sugar and nucleotide sugar metabolism as well as the pentose phosphate pathway, as shown by heat map and metabolic pathway enrichment analysis (MSEA) representations (Fig. 4A and B). The effects of MSC mitochondria and TMZ treatment were found to be additive in many instances, as for example for citrate and malate, glucose 6-phosphate and fructose 6-phosphate, and for the nucleotides AMP, GMP, CMP, and UMP, (Fig. 4C; see also Supplementary Fig. S4 for additional metabolites). Reciprocally, we addressed whether cotreatment of GSCs with MSC mitochondria and TMZ altered their use of TCA cycle–related metabolic substrates, as assayed by Biolog Mitoplates. Important differences were indeed found in the use of specific metabolites, notably *cis*-aconitate and L-malate (Supplementary Fig. S5). Overall, our data demonstrated significant changes in several GSC metabolic pathways, notably the TCA cycle and nucleotide production, following acquisition of MSC mitochondria and TMZ treatment.

### MSC Mitochondria Rewire Metabolic Pathways in GSCs

**FIGURE 5:**
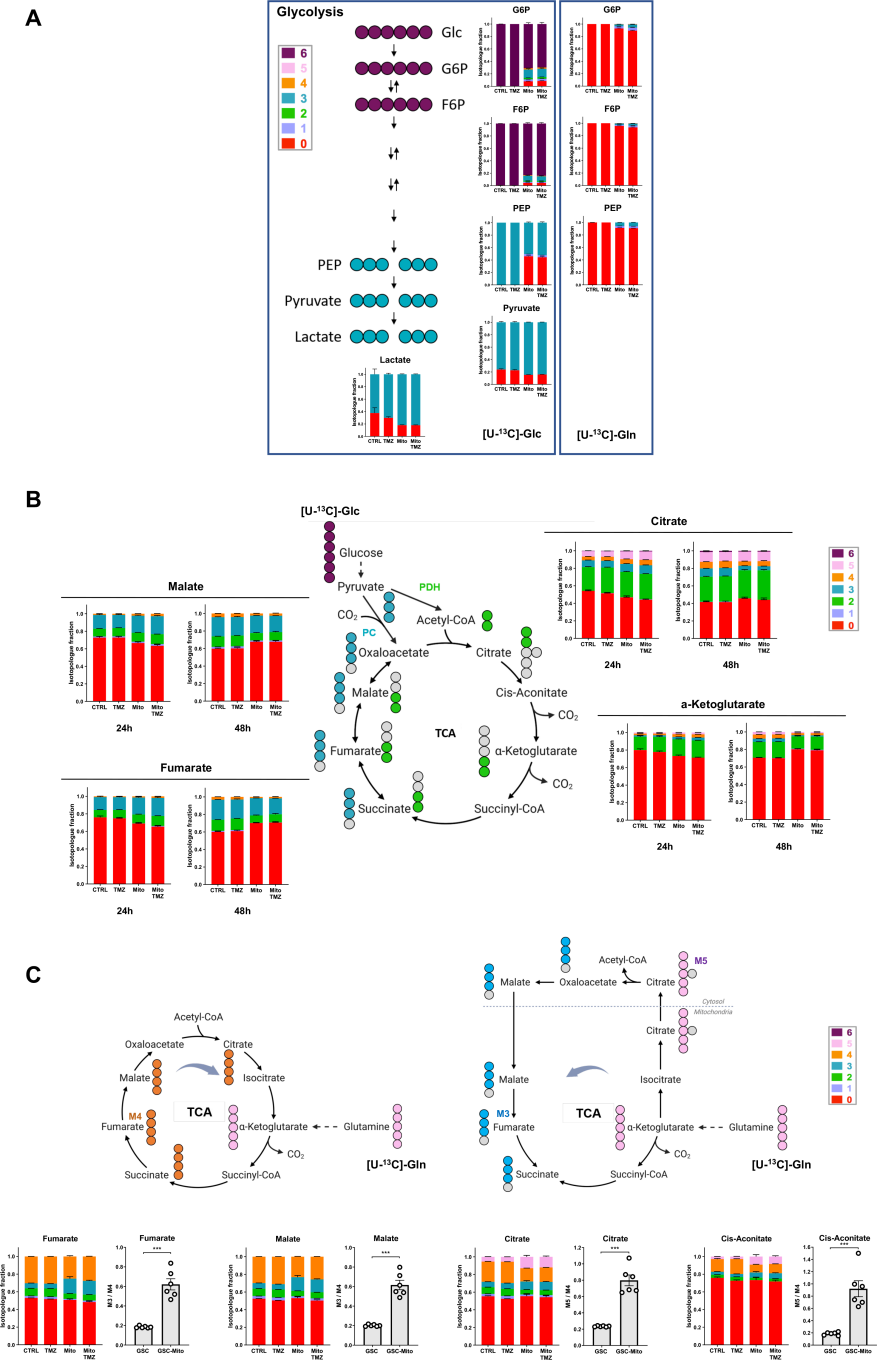
MSC mitochondria modify metabolite fluxes in GSCs. [U-^13^C]-glucose and **[**U-^13^C]-glutamine isotope profiling of GSCs with and/or without MSC mitochondria and treated or not with TMZ (48 hours; *n* = 3). **A,** Glycolysis intermediates. **B,** Isotopologues of TCA cycle metabolites from ^13^C-glucose (24 and 48 hours). **C,** Isotopologues of TCA cycle metabolites from ^13^C-glutamine (48 hours). M3/M4 and M5/M3 ratios of isotopologues in GSCs with/out MSC mitochondria. Mean + SEM. Two-tailed unpaired *t* tests; ***, *P* < 0.001. Colors refer to the number of carbon originating from [U-^13^C]-glucose (A, B) and [U-^13^C]-glutamine (C).

To better characterize the dynamics of metabolic alterations in GSCs following the acquisition of MSC mitochondria, we performed isotope-profiling experiments by using as substrate [U-^13^C]-glucose (Fig. 5A and B) and [U-^13^C]-glutamine (Fig. 5C). As expected, monitoring of [U-^13^C]-glucose metabolism showed the generation of the M+6 isotopologue glycolysis intermediates glucose-6P (Glc6P) and fructose-6P (Fru6P) and of the M+3 isotopologue metabolites PEP, pyruvate, and lactate (Fig. 5A). Interestingly, acquisition of MSC mitochondria by GSCs generated additional glucose-6P and fructose-6P isotopologues, notably M+3 isotopologues (13% and 8%, respectively), as observed at 48 hours. The fraction of PEP directly deriving from [U-^13^C]-glucose was also highly reduced, from 100% in control GSCs to 52% in GSCs with MSC mitochondria, which suggested that reversible glycolysis occurred in GSCs with acquired mitochondria (Fig. 5A).

[U-^13^C]-glucose contributed to the generation of the TCA cycle intermediates citrate, alpha-ketoglutarate, fumarate, and malate, as observed at the two timepoints 24 and 48 hours (Fig. 5B). The detection of both M+2 and M+3 isotopologues indicated that both pyruvate decarboxylation to acetyl-CoA by pyruvate dehydrogenase and carboxylation of pyruvate to oxaloacetate by pyruvate carboxylase were effective in GSCs. At the 24 hours timepoint, GSCs with MSC mitochondria produced a higher percentage of ^13^C-labeled metabolites than control GSCs (e.g., 33% vs. 25%, for fumarate isotopologues), which suggested that MSC mitochondria induced higher TCA turnover from [U-^13^C]-glucose. This pattern, however, unexpectedly changed at 48 hours. While the proportion of ^13^C-labeled metabolites kept increasing in control GSCs (e.g., +38% and +32%, for fumarate and α-ketoglutarate, respectively), in contrast it decreased in GSCs with MSC mitochondria (−11% and −36%, respectively), suggesting that a carbon source other than [U-^13^C]-glucose was preferentially used for anapleurosis in these conditions (Fig. 5B).

To determine whether glutamine was used as an alternative carbon source in GSCs with acquired MSC mitochondria, cultures were performed with [U-^13^C]-glutamine. Incubation of GSCs with [U-^13^C]-glutamine showed active oxidative glutaminolysis as it generated a high fraction of M+4 isotopologue TCA cycle intermediates including fumarate, malate, citrate, and *cis*-aconitate (Fig. 5C). The acquisition of MSC mitochondria by GSCs deeply altered this isotopologue pattern. The proportion of M+3 isotopologues of fumarate and malate increased at the expense of M+4 isotopologues, as shown by the increased M3/M4 ratios (Fig. 5A). Likewise, increased proportions of M+5 isotopologues of citrate and *cis*-aconitate were detected at the expense of the corresponding M+4 isotopologues in GSCs with MSC mitochondria. These metabolic alterations indicated that the acquisition of MSC mitochondria triggered reductive carboxylation in GSCs at the expense of oxidative glutaminolysis.

Supply of GSCs with [U-^13^C]-glutamine also triggered a small, but detectable, amount of Glc6P, Fru6P, and PEP glycolytic compounds with ^13^C originating from glutamine, when GSCs had acquired MSC mitochondria, as observed at 48 hours (Fig. 5A). This observation suggested that gluconeogenesis from glutamine was also occurring in GSCs with MSC mitochondria.

Overall, our data indicated that acquisition of MSC mitochondria induces metabolic rewiring in GSCs, supported by higher glutamine usage. Importantly, these metabolic changes persisted when GSCs were further treated with TMZ.

### Higher Orotate Turnover Supports the Mitochondria-dependent Resistance of GSCs to TMZ

**FIGURE 6:**
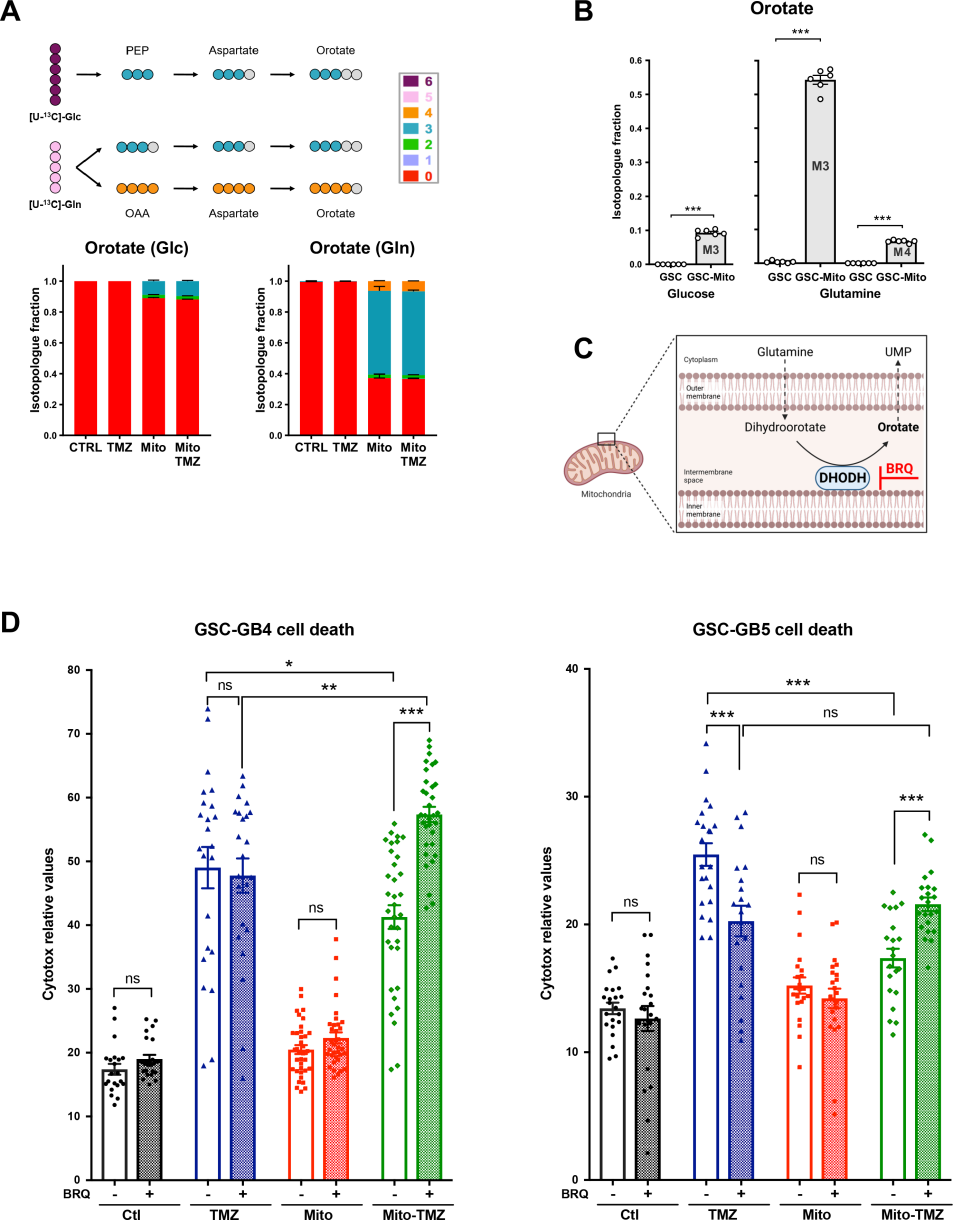
MSC mitochondria induce a higher orotate turnover in GSCs which supports resistance to TMZ. **A** and **B,** [U-^13^C]-glucose and [U-^13^C]-glutamine orotate isotope profiling in GSCs with and/or without MSC mitochondria and treated or not with TMZ (48 hours; *n* = 3). Mean + SEM. Statistical analysis by Student *t* test. ***, *P* < 0.001. **C,** Schematic of BRQ inhibition of DHODH-dependent orotate production (BioRender). **D,** Effect of BRQ (100 nmol/L) on the survival of GSCs (GB4 and GB5, from 2 donors) to TMZ treatment (50 μmol/L) following acquisition of MSC mitochondria (3 MSC donors for each GSC). Two-way ANOVA, *, *P* < 0.05; **, *P* < 0.01; ***, *P* < 0.001; *n* = 4, each dot represents a culture well.

Isotope profiling revealed a dramatic effect of exogenous mitochondria acquisition on orotate turnover in GSCs. At the 48 hours timepoint, a high flux of orotate production was observed from [U-^13^C]-glutamine, with a major fraction of M+3 orotate isotopologue (54%) and a smaller fraction of M+4 orotate isotopologue (6%), whereas no orotate turnover was detected in control GSCs (Fig. 6A and B). Orotate is a precursor of UMP and pyrimidines. It is produced at the mitochondrial membrane through dihydroorotate dehydrogenase (DHODH) activity. To determine whether the increased orotate turnover observed in GSCs after acquisition of MSC mitochondria was instrumental in GSC resistance to TMZ, DHODH-mediated orotate production was blocked by Brequinar (BRQ), a commercially available inhibitor of DHODH (Fig. 6C). BRQ had no detectable effect on GSC survival in control conditions (CTL), after TMZ treatment (TMZ), or after acquisition of MSC mitochondria (Mito; Fig. 6D, left). However, BRQ reversed the effect of MSC mitochondria on GSC cell death in response to TMZ (Mito-TMZ). GSC cell death increased 1.39-fold, restoring TMZ sensitivity to even higher levels than those observed with control GSCs (Fig. 6D, left, compare Mito-TMZ with TMZ in BRQ conditions). We confirmed with GSCs isolated from another patient the supporting role of orotate for mitochondria-induced TMZ resistance (Fig. 6D, right, GSC-GB5). Of note, GB5 GSCs were of a different subtype (non-mesenchymal) compared with GB4 (mesenchymal). Similar to GSC-GB4, MSC mitochondria reduced TMZ-induced cell death in GSC-GB5 (31.20%). The sensitivity of GSC-GB5 to BRQ was found to be different from that of GSC-GB4, as cotreatment of GSC-GB5 with BRQ and TMZ resulted in decreased cell death, compared with TMZ alone. Nevertheless, as with GSC-GB4, in GSC-GB5 with acquired MSC mitochondria, cotreatment with BRQ/TMZ increased cell death (1.24-fold), which was restored to levels found in control GSCs (Fig. 6D, right, compare Mito-TMZ with TMZ in BRQ conditions).

Altogether, these data showed that orotate production constitutes a metabolic dependency for chemoresistant GSCs following acquisition of MSC mitochondria, and that mitochondria-dependent TMZ resistance could be reversed because of the synthetic lethality of TMZ and DHODH inhibition.

### GBM Express Higher Levels of Nucleotides at Relapse after TMZ Treatment

**FIGURE 7:**
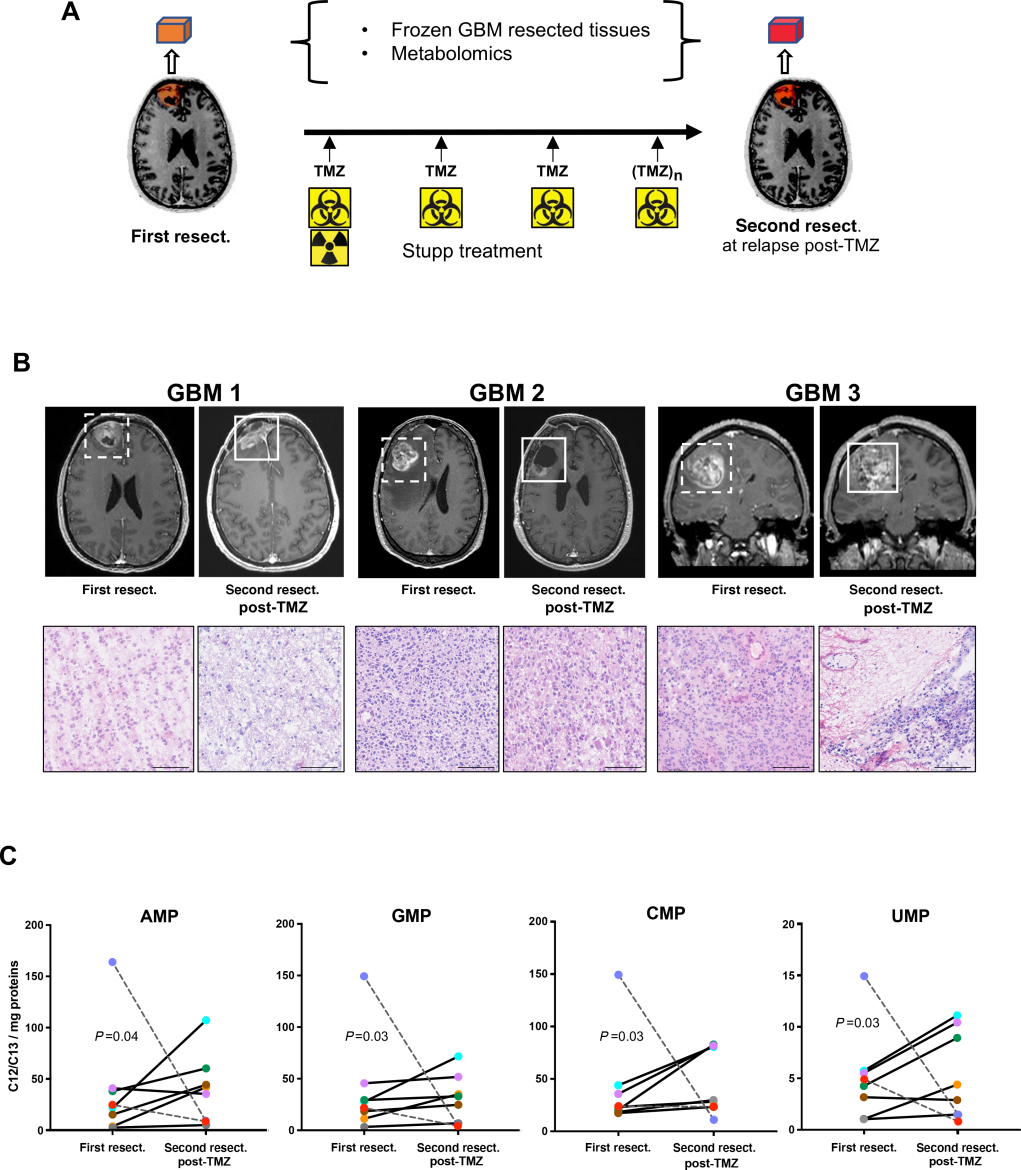
Metabolomics analysis of resected GBM from patients, pre- and post-TMZ treatment shows increased nucleotides concentrations. **A,** Metabolomics analysis were performed on GBM resected tumors from 8 patients, before treatment and after radiotherapy and several cycles (3 to 13) of TMZ treatment. **B,** MRI profiles and H&E-stained tissue sections of the analyzed GBM tumors, before and after TMZ treatment. Tumor areas at first and second resections are framed. Scale bar, 100 μm. **C,** Mass spectrometry metabolomics analysis of the resected GBM. The C12/C13 metabolite ratios normalized to tissue protein concentrations are indicated. Out of the 8 patients, 6 followed a similar trend (solid lines), while 2 followed an opposite trend (dotted lines). Statistical analysis by ratio paired Student *t* test (AMP, GMP, UMP) and Wilcoxon matched-pairs signed-rank test (CMP) on metabolomics data from the 6 patients.

To check the clinical relevance of our *in vitro* findings, we further compared metabolite levels in patient tumors at first resection prior to treatment and at the second resection at relapse after TMZ treatment (Fig. 7A). Although most patients do not undergo a second resection after Stupp treatment, we were able to obtain frozen resected GBM tissues from 8 patients, as well as clinical and MRI data (Materials and Methods and Fig. 7B). Metabolomics analysis was performed on these GBM tissues, whose representative hematoxylin and eosin (H&E)-stained sections are shown (Fig. 7B). The levels of metabolites measured in these tumors (related to protein mass) differed between patients and pairwise analysis did not identify a definitive pattern for most of the metabolites measured (Supplementary Fig. S6), which could be explained by the many parameters that could influence metabolite concentrations in these tissues. However, quite strikingly, 6 of the 8 patients followed a similar trend for the four ribonucleoside monophosphates AMP, GMP, CMP, and UMP, whose levels were all increased at relapse after TMZ treatment, indicative of greater transcriptional activity. Furthermore, these increased nucleotide concentrations were consistent with our *in vitro* metabolomics data (Fig. 4C), suggesting that mitochondria transfer may have promoted GBM relapse after TMZ treatment in these patients.

## Discussion

Intercellular interactions support the rapid adaptation of cells to stress conditions. In tumors, interactions between cells of the microenvironment and cancer cells promote chemoresistance. In particular, mitochondria transfer from MSCs has been reported as one of the means allowing cancer cells to escape therapy. Consistently, the presence of MSCs in GBM tumors is associated with a poor prognosis (39, 40, 42, 43). However, the biological mechanisms for this chemotherapy resistance have not been determined so far. This prompted us to question the impact of MSC mitochondria transfer to GSCs on GBM tumor progression.

By using both large-scale analyses and our Mitoception protocol, which allows quantitative transfer of exogenous mitochondria (49, 53, 54), we provide evidence that MSC mitochondria induce resistance of GSCs to TMZ and that this resistance is supported by alterations in GSC metabolism. Indeed, the major advance of our study was brought by the analysis of the metabolic fluxes of GSCs, using [U-^13^C]-glucose and [U-^13^C]-glutamine as substrates. This analysis revealed that MSC mitochondria trigger a major metabolic switch in GSCs, by promoting glutamine utilization and by favoring reductive carboxylation over glutaminolysis. MSC mitochondria also increased glutamine-dependent orotate turnover in GSCs, in direct connection to respiratory chain and DHODH activities and to downstream pyrimidine biosynthesis. One of the major findings of our study is that the increased resistance of GSCs to TMZ conferred by acquired MSC mitochondria is supported by this higher turnover of orotate, since inhibition of orotate production by BRQ led to synthetic lethality with TMZ treatment. These results were obtained with GSCs, of both mesenchymal and non-mesenchymal subtypes derived from 2 donors, and with mitochondria isolated from MSCs of 3 donors.

Several studies on both normal and cancer cells reported increased OXPHOS following acquisition of exogenous mitochondria [see reviews (25, 47)]. We also observed an increase in GSC OXPHOS following acquisition of MSC mitochondria, which was maintained upon TMZ treatment. Enhanced OXPHOS has been previously associated to survival of damaged cells and proliferation of cancer cells, as shown notably in damaged cardiomyocytes and in ρ0 cells, which are devoid of mtDNA (50, 57, 58). These effects have been largely attributed to increased ATP production. However, in addition to ATP, effective OXPHOS can also be associated with sustained orotate production, previously shown to be a major trigger of tumor growth (59). The results presented here demonstrate the role of orotate production in the TMZ resistance of GSCs induced by MSC mitochondria as well as the synthetic lethality achieved by cotreatment of GSCs with BRQ and TMZ. Targeting the metabolic reprogramming of cancer cells has been previously proposed as a promising tool for cancer therapy (11, 14, 60, 61). Our own results reveal a targetable metabolic vulnerability in GSCs and raise novel opportunities for future therapeutic strategies. In this context, it is worth noting that inhibition of pyrimidine synthesis was able to impede GSC growth in preclinical models (62–64) and that BRQ as well as other inhibitors of DHODH activity are currently evaluated in clinical trials (65, 66).

Interestingly, these metabolic effects were triggered by the acquisition of small amounts of exogenous MSC mitochondria (0.4% of GSC endogenous mitochondria). These few exogenous mitochondria, however, had the capacity to induce an increase in total mitochondrial levels in GSCs, as demonstrated by an increase in GSC mtDNA concentrations and mitochondrial mass. Similar findings have been previously reported, by us and others, for the breast cancer cell line MDA-MB-231 and for acute myeloid leukemia cells (49, 55). This could therefore reflect a general process, whereby acquired mitochondria have the capacity to trigger endogenous mitochondrial production. Its precise mechanism as well as the fate of the transferred mitochondria remain to be fully investigated.

Our metabolomics study showed that MSC mitochondria altered several GSC metabolic pathways in TMZ-treated GSCs, notably the pyrimidine and purine synthesis pathways. We interrogated whether similar mechanisms might be at play in GBM tissues from patients, at relapse following TMZ treatment. Although secondary surgery is performed in only a small percentage of patients with GBM, we managed to retrieve paired resected GBM tissues for 8 patients. For 6 out of these 8 patients, the levels of the four nucleoside monophosphates, AMP, GMP, CMP, and UMP, were found increased in relapsed versus primary GBMs, consistent with the stimulation of nucleotide synthesis. This trend, which was observed in 75% of the patients with GBM, is in good correlation with our *in vitro* data. It will obviously need to be further confirmed by a larger cohort. Still, it already provides important information on metabolic markers at GBM relapse that could be of primary interest for patient follow-up.

Our isotopic profiling experiments showed that the higher orotate turnover we observed in GSCs relied on the use of glutamine as a substrate. Importantly, higher concentrations of glutamine have been reported in GBM tumors as compared with the surrounding normal brain tissue, with astrocytes as a likely source of glutamine release, so that glutamine uptake was proposed as a hallmark of GBMs (22, 67–69). Our work therefore provides a mechanism by which the detected higher concentrations of glutamine in GBM can support progression of the tumor, through processes involving intercellular mitochondria transfers. Therefore, from a clinical perspective, currently available glutamine (^18^F-FGln) PET imaging (70, 71) could also provide an attractive tool to detect patients relapse after TMZ therapy, in relation to intercellular mitochondria transfers.

We observed that MSC mitochondria increased ROS production in TMZ-treated GSCs. Although ROS contribute to genetic instability and tumor progression, a large increase in ROS production may also lead to therapeutic susceptibility which, however, is often prevented by cancer cells increased antioxidant response (72, 73). Controlling ROS levels in GSCs, for instance by inhibiting SOD2, could therefore be a potential therapeutic strategy for GBM progressing on TMZ therapy, the relevance of which definitely requires further research.

Because of the presence of a complex microenvironment, overcoming GSC resistance to TMZ *in vitro* obviously does not equate to overcoming GBM resistance to TMZ *in vivo*. The technical emergence of minibrains and the possibility to engraft GBM cells as well as MSCs should allow addressing these issues in the near future (74, 75). It could allow monitoring the dynamics of TNT formation between MSCs and GSCs *in vivo* and, besides, identify other possible cellular cargoes trafficking within these TNTs, which could act in concert with mitochondria in supporting GBM progression.

Another important question raised by our study is whether other cells in the glioma microenvironment may contribute to GBM chemoresistance through mitochondria transfer. Supporting this hypothesis, astrocytes have been reported to transfer mitochondria through TNTs to GBM cells and, besides, to confer cisplatin resistance on noncancerous neurons (76, 77). Our own preliminary data indicate that astrocyte mitochondria may also confer GSC resistance to TMZ. It will be of interest to determine whether the acquired resistance relies on mechanisms similar to those we described here for MSC mitochondria. Further investigation of these mechanisms as well as the possible role of other cell types in the vicinity of GBM tumors, such as neurons and endothelial cells, is definitely needed before designing novel effective therapies for GBM.

### Limitations of the Study

We analyzed here a cohort of 8 patients who had undergone Stupp clinical protocol after their first GBM resection, meaning that these patients were initially treated simultaneously with radiotherapy and TMZ, before being treated with multiple cycles of TMZ alone. Therefore, the metabolic modifications we have reported in the GBM resected tumors at relapse from TMZ might also partly reflect consequences of radiotherapy.

In many studies performed on patient tumors, such as the one we performed here, cells are not isolated before analysis and some contribution of the stroma to data acquisition cannot be avoided. For transcriptional analysis, this caveat can now be solved by single-cell RNA sequencing. Fortunately, single-cell metabolic analysis is also emerging as an accessible tool that will undoubtedly provide crucial information and advance the field (78–80).

## Supplementary Material

Video 1 Cocultures of red MitoTracker-labeled MSCs and green CellTracker-labeled GSCs

Supplementary Materials and Methods TMZ dose-response assay
Caspase-GloⓇ 3/7 assay
Cytotox assay
Extracellular Flux Assays
Cell Proliferation
Western Blot
Flow Cytometry
Metabolite usage Mitoplates
Mass Spectrometry Quantification
13C stable isotope tracing experiments
Preparation and quantification of MSC mtDNA in GSCs
Imaging

Figure S1 Experimental design and timeline

Figure S2 Quantification of mitochondrial DNA (mtDNA) following transfer of MSC mitochondria to GSCs (A) Detection of MSC mtDNA in GSCs. MSC mtDNA concentrations are expressed relative to GSC mtDNA. Mean with SEM, t test with Welch correction, *p < 0.05. (B) Total mtDNA concentrations in GSCs at different time points after MSC mitochondria transfer, expressed relative to GSC genomic DNA. Mean + SEM and multiple t tests, *p < 0.05.

Figure S3 Effects of MSC mitochondria on GSCs metabolic response to TMZ (A, B) GSC mitochondrial mass and ROS production. FACS analysis of MitoTracker and MitoSox-labeled GSCs after the acquisition of MSC mitochondria and TMZ treatment (24 hr). (A) Mitochondrial mass. (B) ROS production. (A, B) Representative experiments and relative MFI values (mean ± SEM; n=3). One-way ANOVA, *p < 0.05. (C) Expression of Cytochrome c oxidase IV (COX IV). Representative Western blot with the corresponding membrane (MW markers in kDa) as shown in Fig. 3H.

Figure S4 Metabolites produced by GSCs following MSC mitochondria acquisition and TMZ treatment

Figure S5 MSC mitochondria modify the usage of metabolites by GCSs in response to TMZ CTRL Mito TMZ CTRL Mito Mito TMZ (A) Metabolic substrate consumption of GSCs expressed as metabolite initial consumption rates (Biolog MitoPlates). Mean values ± SEM (4 independent experiments). (B) Production of cis-aconitate, succinate and L-malate expressed as a percentage of all TCA metabolites produced in each experimental condition. Tukey boxplots with one-way ANOVA, *p < 0.05, ***p < 0.001.

Figure S6 Metabolite detection in resected GBM from 8 patients, at 1rst resection and at 2nd resection post-TMZ treatment. For each metabolite, the C12/C13 ratios normalized to tissue protein concentrations are indicated.
