# Supplementary material for: Mitochondria Transfer from Mesenchymal Stem Cells Confers Chemoresistance to Glioblastoma Stem Cells through Metabolic Rewiring
Source: Cancer Res Commun. 2023 Jun 14;3(6):1041–56. doi: 10.1158/2767-9764.CRC-23-0144 (PMC10266428; doi:10.1158/2767-9764.CRC-23-0144)
Supplement: Supplementary Materials and Methods — TMZ dose-response assay Caspase-GloⓇ 3/7 assay Cytotox assay Extracellular Flux Assays Cell Proliferation Western Blot Flow Cytometry Metabolite usage Mitoplates Mass Spectrometry Quantification 13C stable isotope tracing experiments Preparation and quantification of MSC mtDNA in GSCs Imaging [file crc-23-0144-s02.pdf]

SUPPLEMENTARY MATERIALS AND METHODS

TMZ dose-response assay

Cells were seeded in 96-well plates coated with poly-D-lysine (0.1 mg/mL, Sigma-Aldrich P7280), at a concentration of $2x10^4$ cells/well. Cells were treated with TMZ (0 – 400 µM, Sigma-Aldrich T2577), prepared by serial dilutions in the culture medium with a constant DMSO concentration (0.1%). After 5 days of treatment, cells were fixed with paraformaldehyde (4%, Euromedex 15714) and labelled with Hoechst 33342 (5 mg/mL, Invitrogen H3570). Cell numbers were determined by stained nuclei counting (Thermo Scientific Cellomics BioApplications-Image).

Caspase-Glo® 3/7 assay

Caspase-Glo® 3/7 assays (Promega G8091) were carried out according to the manufacturer's instructions. Briefly, cells were seeded in white-walled 96-well plates coated with poly-D-lysine (0.1 mg/mL, Sigma-Aldrich P7280), at a concentration of $2x10^4$ cells/well. Cells were treated with TMZ (50 µM, Sigma-Aldrich T2577) or the equivalent DMSO concentration (0.1%). After 5 days of treatment, the Caspase-Glo® 3/7 reagent was added to each well (1:1). Plates were homogenized for 30 sec using a plate shaker and then incubated for 30 min at room temperature. Luminescence was measured for 1 sec using the Mithras LB 940 plate reader and the MikroWin 2000 software (Berthold Technologies). After background luminescence subtraction, luminescence was normalized to the cell number per well, determined by Hoechst 33342 (5 mg/mL, Invitrogen H3570)-stained nuclei counting (Thermo Scientific Cellomics BioApplications-Image).

Cytotox assay

Cells were seeded in 96-well plates coated with poly-D-lysine (0.1 mg/mL, Sigma-Aldrich P7280), at a concentration of $2x10^4$ cells/well. Cells were treated with TMZ (50 µM, Sigma-Aldrich T2577) or the equivalent DMSO concentration (0.1%). After 5 days of treatment, Incucyte® Cytotox red dye (250 nM, Sartorius 4633) was added in each well (1:1). The plates were placed in the Incucyte® Live-Cell Analysis System. Phase contrast and red fluorescence (400 ms) acquisitions were performed at 10x magnification after 2 hours. The numbers of Cytotox labeled cells were normalized either to GSC cell confluence or cell number. Alternatively, nuclei were labeled with Hoechst and the numbers of both Cytotox-stained cells and Hoechst-stained nuclei were evaluated by Celigo imaging cytometry.

Extracellular Flux Assays

For all extracellular flux assays, GSCs were seeded at a density of 4 x 10^4 cells per well on Seahorse XFe96 culture microplates coated beforehand with poly-lysine (0.1 mg/ml) (Sigma-Aldrich P7280) and laminin (10 ug/ml) (Sigma-Aldrich L2020). The assay plates were spin-seeded for 5 min at 1,200 rpm and incubated at 37°C with CO_2 for 48 hr prior to performing the assay on the Seahorse Bioscience XFe96 (RRID:SCR_019545). Before the assay, cells were incubated in the assay medium for 1 hr at 37°C without CO_2. Sensor plates were calibrated overnight in XF calibration buffer at 37°C in atmospheric conditions without CO_2 supplementation.

Oxygen Consumption Rate (OCR) Assays

OCR measurements were performed in XF media (non-buffered DMEM) supplemented with glucose (10 mM) (Seahorse XF 103577-100), L-glutamine (2 mM) (Gibco 25030024) and sodium pyruvate (1 mM) (Seahorse XF 103578-100), under basal conditions and in response to mitochondrial inhibitors: oligomycin (1 uM) (Sigma-Aldrich O4876), FCCP (1 uM) (Sigma-Aldrich C2920), rotenone (100 nM) (Sigma-Aldrich R8875) and antimycin A (1 uM) (Sigma-Aldrich A8674).

Extracellular Acidification Rate (ECAR) Assays

ECAR measurements were performed in XF media supplemented with L-glutamine (2 mM) (Gibco 25030024) and sodium pyruvate (1 mM) (Seahorse XF 103578-100), in response to glucose (10 mM) (Seahorse XF 103577-100), oligomycin (1 uM) (Sigma-Aldrich O4876), oxamate (75 mM) (Sigma-Aldrich O2751) and 2-deoxyglucose (2-DG, 100 mM) (Sigma-Aldrich D8375).

For each biological replicate, at least four technical replicates were prepared. Oxygen consumption rates (OCR, pMoles/min) and extracellular acidification rates (ECAR, mpH/min) were measured every 6 min (3-minute mix and 3-minute measurement), 3 times for each condition. All measures were normalized to the number of cells counted in each well of the Seahorse XFe96 culture microplates at the end of the SeaHorse experiments, on the basis of Hoechst (Invitrogen H3570) nuclei labeling and automatic counting (Thermo Scientific Cellomics BioApplications-Image). The data were processed with the Agilent Wave software (RRID:SCR_014526).

Cell Proliferation

Cell growth of GSCs upon acquisition of MSC mitochondria was determined by automated cell counting. In brief, GSCs were seeded at a density of 4 x 10^4 cells per well in 96-well plates, the day following Mitoception. Forty-eight hours later, cells were fixed in 4% formaldehyde (Euromedex 15714) and stained with Hoechst 33342 (5 mg/ml, Invitrogen H3570). Cell numbers were determined on the basis of the stained nuclei which were quantified automatically (Thermo Scientific Cellomics BioApplications-Image).

Western Blot

For GSC protein samples preparation, GSCs (2 x 10^5) were collected directly in Laemmli buffer (2X) (Bio-Rad 161-0747). After heating (95°C) for 5 minutes, protein samples (equivalent to 4 x 10^4 cells) were run using Bio-Rad Mini-Protean TGX 4-15% gradient gels (Bio-Rad 4561083) and transferred onto PVDF membranes (Bio-Rad 1704157). Membranes were blocked for 30 minutes in 5% skimmed milk in TBST at room temperature and incubated with primary antibodies overnight in 1% skimmed milk in TBST according to manufacturer suggested primary antibody dilutions. Membranes were then incubated with HRP-conjugated secondary antibodies at a 1:10,000 dilution, developed using Millipore ECL (WBKLS0500) and quantified using the BioRad ChemiDoc XRS instrument. Antibodies directed against COXIV (GeneTex Cat# GTX628901, RRID:AB_2888077), SOD2 (GeneTex Cat# GTX116093, RRID:AB_10624558) and β-actin (Abcam Cat# ab8226, RRID:AB_306371) were used.

Flow Cytometry

GSCs (2.5 x 10^5 cells) were dissociated to single cells by pipetting prior to the labeling with MitoTracker Deep Red FM (250 nM, Molecular Probes, M22426), MitoSOX^{TM} Red mitochondrial superoxide indicator (5 uM, Molecular Probes, M36008) and Zombie violet (1X, BioLegend, BLE423113). Fluorescence-activated cell sorting was performed on a Gallios Beckman Coulter Flow Cytometer (RRID:SCR_019639) and analysis was done with the GalliosTM Kaluza (RRID:SCR_016700) software.

Metabolite usage Mitoplates

GSCs (8 x 10^4 cells/well) were seeded in 96-well MitoPlate^{TM} S-1 Microplates (Biolog). MitoPlates^{TM} S-1 contain 3 sets of wells pre-coated with either cytoplasmic or mitochondrial substrates. Saponin (50 ug/ml) was used to permeabilize the GSCs. Substrate usage was determined on the basis of the initial usage rate measured, in each well, at 37°C with a Biolog OmniLog instrument.

Mass Spectrometry Quantification

The analyses were carried out on an IC-MS platform with a liquid anion exchange chromatography Dionex™ ICS-5000+ Reagent-Free™ HPIC™ (Thermo Fisher Scientific™, Sunnyvale, CA, USA) system, coupled to a Thermo Scientific™ LTQ Orbitrap Velos™ mass spectrometer (Thermo Fisher Scientific, San Jose, CA, USA) equipped with a heated electrospray ionization probe.

Analytes were separated within 50 min, using a linear KOH gradient elution applied to an IonPac AS11-HC column (250 x 2 mm, Dionex) equipped with an AG11-HC guard column (50 x 2 mm, Dionex) at a flow rate of 0.38 ml/min. The gradient program was the following: equilibration with KOH (7 mM) for 1.0 min; then KOH ramp from 7 to 15 mM, 1–9.5 min; constant concentration 10.5 min; ramp to 45 mM in 10 min; ramp to 70 mM in 3 min; ramp to 100 mM in 0.1 min; constant concentration 8.9 min; drop to 7 mM in 0.5 min

and equilibration at 7 mM KOH for 7.5 min. The column and autosampler temperatures were thermostated at 25°C and 4°C, respectively. The injected sample volume was 15 µl. Measures were performed in triplicates from separate cultures.

Mass detection was carried out in a negative electrospray ionization (ESI) mode at a resolution of 60 000 (at 400 m/z) in full-scan mode, with the following source parameters: the capillary temperature was 350 °C, the source heater temperature, 300 °C, the sheath gas flow rate, 50 a.u. (arbitrary unit), the auxiliary gas flow rate, 5 a.u., the S-Lens RF level, 60%, and the source voltage, 2.75 kV. Data acquisition was performed using Thermo Scientific Xcalibur software. Metabolites were determined by extracting the exact mass with a tolerance of 5-10 ppm. Data processing: TraceFinder 4.1 software. Raw measures were normalized to cell numbers.

For the Heatmap of GSC metabolites, C12/C13 ratios were represented for each metabolite and for all 4 conditions (n=6 independent measures). Considering that C12/C13 metabolite ratios follow both normal and non-normal distributions, values for each metabolite were normalized as follows: (value – minimal value)/(maximal value – minimal value). This preserved the distribution patterns for each individual metabolite and allowed their comparison on similar scales. Final row max = 1 and row min = 0.

Metabolic Set Enrichment Analysis (MSEA) analysis was performed using the ''Pathway Analysis'' module on MetaboAnalyst (RRID:SCR_015539) 5.0 (www.metaboanalyst.ca ; (50)). Data were range-scaled (mean-centered and divided by the range of each variable) and mapped to the human KEGG pathway library using the global test and relative-betweenness centrality algorithms. Pathway impact reflects the matched number of metabolites relative to the total number of metabolites in a given pathway, as well as their relative importance in that pathway.

^{13}C stable isotope tracing experiments

Samples were evaporated to dryness in a Speedvac Savant SC250Exp (Thermo Fisher Scientific), and resuspended in 200 µL of water before analysis. Debris were removed by centrifugation at 10,000 g for 5 min, and samples were then analyzed by LC-HRMS. Metabolites were analyzed with an ICS5000+, ion chromatography system (Dionex) coupled to an LTQ Orbitrap Velos mass spectrometer (Thermo Fisher Scientific). Metabolites were separated on an ionic chromatography column IonPac AS11 (250 × 2 mm i.d.; Dionex). Mobile phase used was a gradient of KOH at a flow rate of 350 µL/min. Mobile phase varied as follows: 0 min: 0.5 mM, 1 min: 0.5 mM, 9.5 min: 4.1 mM, 14.6 min: 4.1 mM, 24 min: 9.65 mM, 31.1 min: 90 mM, and 43 min: 90 mM. The column was then equilibrated for 5 min at the initial conditions before the next sample was analyzed. The injection volume was 15 µl. MS analyses were performed with a heated electrospray ionization probe in negative FTMS mode at a resolution of 60,000 (at 400 m/z) in full-scan mode, with the following source parameters: capillary temperature, 350 °C; source heater temperature,

300 °C; sheath gas flow rate, 50 a.u. (arbitrary unit); auxiliary gas flow rate, 5 a.u.; S-Lens RF level, 60%; and source voltage, 3.5 kV. Isotopic clusters were determined by extracting the exact mass of all isotopologues with a tolerance of 5 ppm with Tracefinder software (Thermo Fisher Scientific). Carbon isotopologue distributions (CIDs) were calculated after correction of raw MS data using IsoCor software.

Preparation and quantification of MSC mtDNA in GSCs

DNA from GSCs was prepared using a TRIzol-based protocol as previously described (57). Briefly, GSCs (5 x 10^5 cells) were homogenized in 700 µL TRIzol reagent. After addition of chloroform, the upper aqueous phase was recovered and DNA was precipitated in the presence of glycogen (Invitrogen 10814010) and isopropyl alcohol. Mitochondrial DNAs were quantified by PCR by using the SYBR Green Master PLUS Mix (Roche 03515885001) and the LightCycler 480 instrument (Roche, Meylan, France, RRID:SCR_020502), with the following program: 10 min at 95 °C, 50 cycles of 10 s at 95 °C, 15 s at 67 °C and 15 s at 72 °C. Total mitochondrial DNA (mtDNA) was quantified by amplifying a DNA domain within the D-loop of mtDNA by using the following primers: Universal-F: 5'- TTA ACT CCA CCA TTA GCA CC -3'; Universal-R: 5'- GAG GAT GGT GGT CAA GGG A -3' (58). To specifically amplify mtDNA from MSCs (donor MSC119), the following set of primers was used: MSC-F: 5’-AAG CAA GTA CAG CAA TCA ACC CC-3’; MSC-R: 5’-TTA AGG GTG GGT AGG TTT GTA GC-3’ (57). To increase the specificity of the MSC primers for MSC mtDNA, by further diminishing their binding capacity to GSC mtDNA, an additional mismatch was introduced in the MSC primer sequences. This mismatch was found to decrease the efficacy of the initial PCR amplification cycles of the MSC mtDNA. This technically-based difference in the PCR cycle numbers (5.5 cycles) was taken into account in the calculation of the amount of MSC mtDNA in the GSCs following the transfer of MSC mitochondria by Mitoception.

Imaging

Cell imaging was performed on GSCs and MSCs respectively labeled, prior to the coculture, with the vital dyes Green CellTracker CMFDA (4 uM, Molecular Probes C2925) and Red MitoTracker CMXRos (500 nM, Molecular Probes M7512). The coculture was performed in αMEM/FCS 5% in 6-well plates. Imaging started 24 hr after the beginning of the coculture. Confocal fluorescence imaging was done on live cells with a Carl Zeiss LSM 5 live duo (LSM 510 META and 5 live) confocal laser system using a Zeiss 40X plan NeoFluar Oil objective. Time-lapse imaging was performed with an IncuCyte® S3 Live-Cell Analysis System (RRID:SCR_019874) with a 10X objective. Pictures were taken every 30 minutes for 72 hours, for Green CellTracker, Red MitoTracker and phase contrast, with acquisition times of 250 ms for the green channel and 400 ms for the red channel. Stacks of images were exported in Tagged Image File Format (TIFF) and treated with the ImageJ (RRID:SCR_003070) image processing program. Background was removed from the green and red channel image sequences by using the Substract Background function (Rolling ball radius: 50

pixels). Phase image sequences were first stabilized (Image Stabilizer Log Applier plugin) (59). The same log transformation coefficients were applied to the green and the red sequences to align the fluorescent images to the phase (Image Stabilizer Log Applier plugin). The colors of the phase images were inverted and all three sequences inverted phase, green and red sequences were merged using the ImageJ Image Calculator command. After treatment, all image sequences were exported as .AVI films (compression: JPEG and Frame Rate: 4 fps).
