# Supplementary figures and images for: Mitochondria Transfer from Mesenchymal Stem Cells Confers Chemoresistance to Glioblastoma Stem Cells through Metabolic Rewiring
Source: Cancer Res Commun. 2023 Jun 14;3(6):1041–56. doi: 10.1158/2767-9764.CRC-23-0144 (PMC10266428; doi:10.1158/2767-9764.CRC-23-0144)

# Figure S1

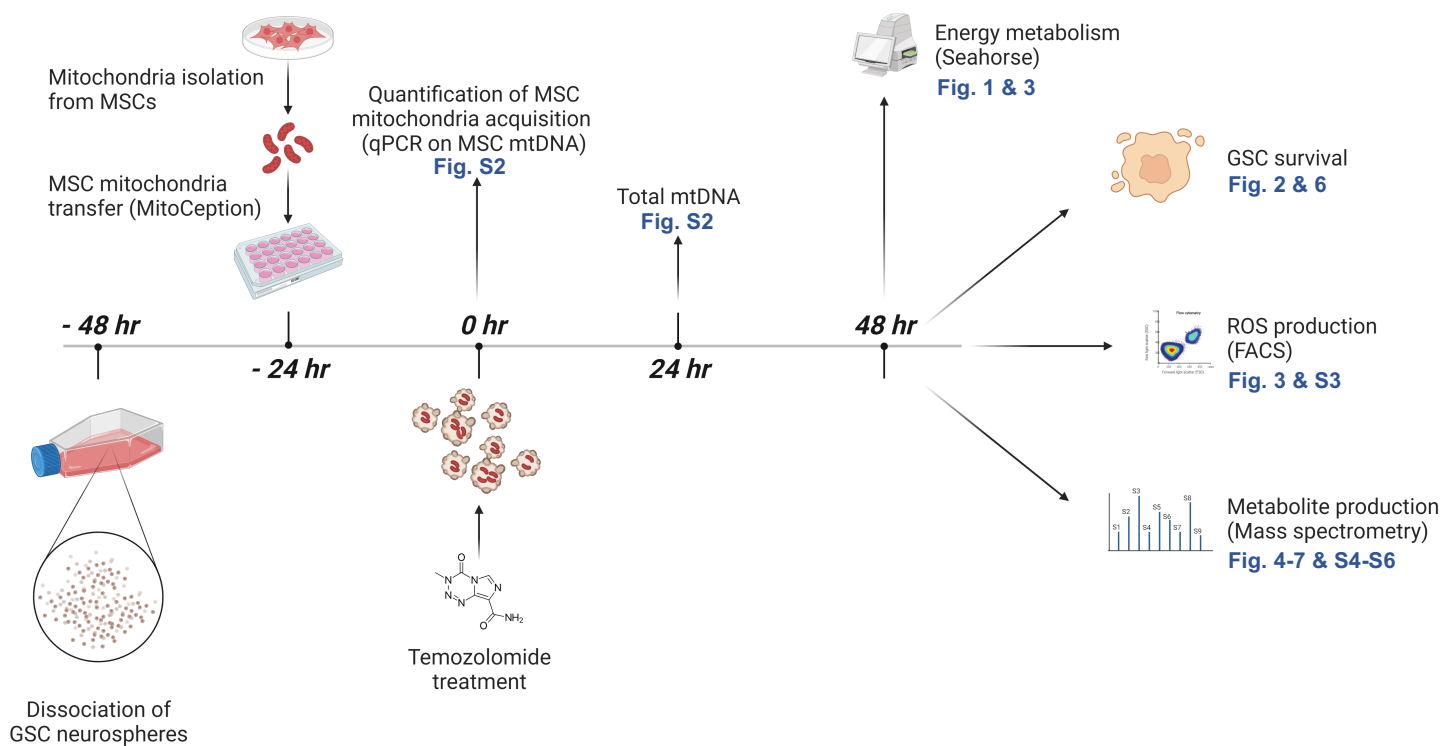

Experimental design and timeline (BioRender)

Supplement: Figure S1 — Experimental design and timeline [file crc-23-0144-s03.pdf]
