# Supplementary material for: Mitochondria Transfer from Mesenchymal Stem Cells Confers Chemoresistance to Glioblastoma Stem Cells through Metabolic Rewiring
Source: Cancer Res Commun. 2023 Jun 14;3(6):1041–56. doi: 10.1158/2767-9764.CRC-23-0144 (PMC10266428; doi:10.1158/2767-9764.CRC-23-0144)
Supplement: Figure S2 — Quantification of mitochondrial DNA (mtDNA) following transfer of MSC mitochondria to GSCs (A) Detection of MSC mtDNA in GSCs. MSC mtDNA concentrations are expressed relative to GSC mtDNA. Mean with SEM, t test with Welch correction, *p < 0.05. (B) Total mtDNA concentrations in GSCs at different time points after MSC mitochondria transfer, expressed relative to GSC genomic DNA. Mean + SEM and multiple t tests, *p < 0.05. [file crc-23-0144-s04.pdf]

Figure S2

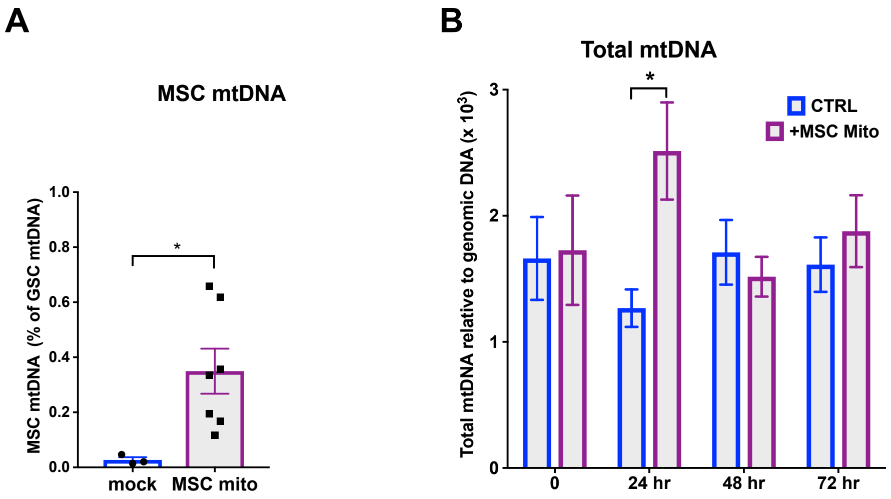

Quantification of mitochondrial DNA (mtDNA) following transfer of MSC mitochondria to GSCs (A) Detection of MSC mtDNA in GSCs. MSC mtDNA concentrations are expressed relative to GSC mtDNA. Mean with SEM, t test with Welch correction, *$p < 0.05$. (B) Total mtDNA concentrations in GSCs at different time points after MSC mitochondria transfer, expressed relative to GSC genomic DNA. Mean + SEM and multiple t tests, *$p < 0.05$.
