# Supplementary material for: Mitochondria Transfer from Mesenchymal Stem Cells Confers Chemoresistance to Glioblastoma Stem Cells through Metabolic Rewiring
Source: Cancer Res Commun. 2023 Jun 14;3(6):1041–56. doi: 10.1158/2767-9764.CRC-23-0144 (PMC10266428; doi:10.1158/2767-9764.CRC-23-0144)
Supplement: Figure S3 — Effects of MSC mitochondria on GSCs metabolic response to TMZ (A, B) GSC mitochondrial mass and ROS production. FACS analysis of MitoTracker and MitoSox-labeled GSCs after the acquisition of MSC mitochondria and TMZ treatment (24 hr). (A) Mitochondrial mass. (B) ROS production. (A, B) Representative experiments and relative MFI values (mean ± SEM; n=3). One-way ANOVA, *p < 0.05. (C) Expression of Cytochrome c oxidase IV (COX IV). Representative Western blot with the corresponding membrane (MW markers in kDa) as shown in Fig. 3H. [file crc-23-0144-s05.pdf]

Figure S3

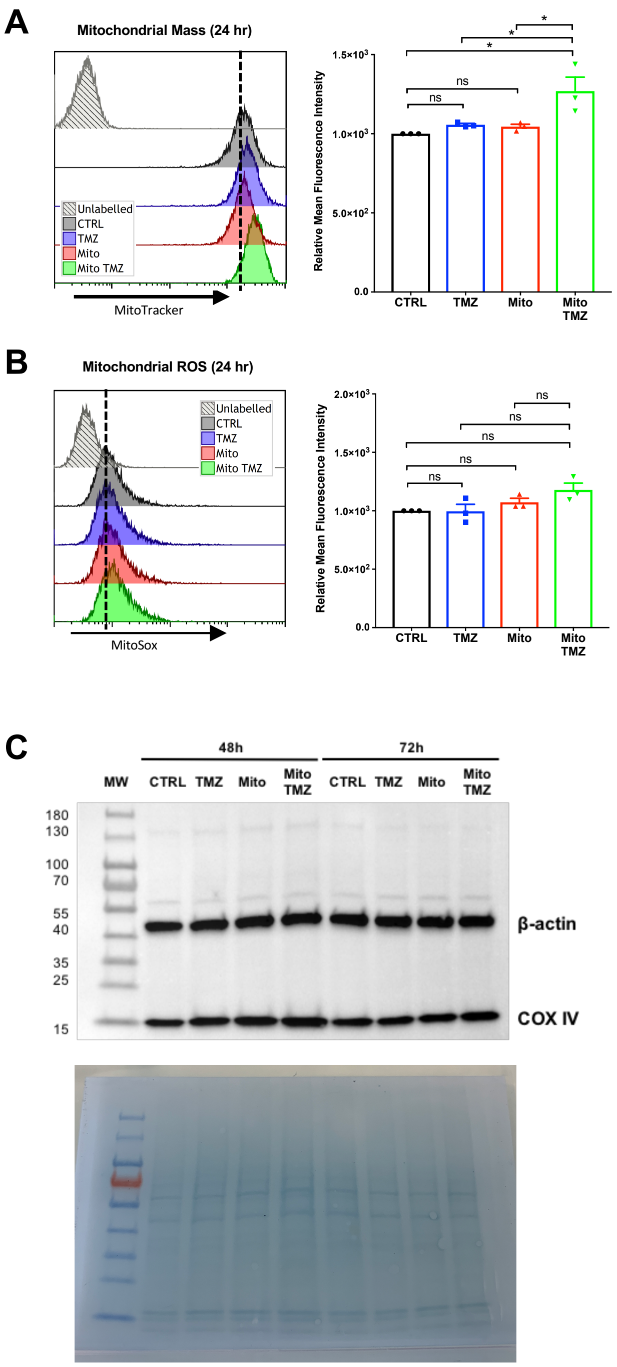

Effects of MSC mitochondria on GSCs metabolic response to TMZ (A, B) GSC mitochondrial mass and ROS production. FACS analysis of MitoTracker and MitoSox-labeled GSCs after the acquisition of MSC mitochondria and TMZ treatment (24 hr). (A) Mitochondrial mass. (B) ROS production. (A, B) Representative experiments and relative MFI values (mean ± SEM; n=3). One-way ANOVA, *$p < 0.05$. (C) Expression of Cytochrome c oxidase IV (COX IV). Representative Western blot with the corresponding membrane (MW markers in kDa) as shown in Fig. 3H.
