# Supplementary material for: Mitochondria Transfer from Mesenchymal Stem Cells Confers Chemoresistance to Glioblastoma Stem Cells through Metabolic Rewiring
Source: Cancer Res Commun. 2023 Jun 14;3(6):1041–56. doi: 10.1158/2767-9764.CRC-23-0144 (PMC10266428; doi:10.1158/2767-9764.CRC-23-0144)
Supplement: Figure S4 — Metabolites produced by GSCs following MSC mitochondria acquisition and TMZ treatment [file crc-23-0144-s06.pdf]

Figure S4

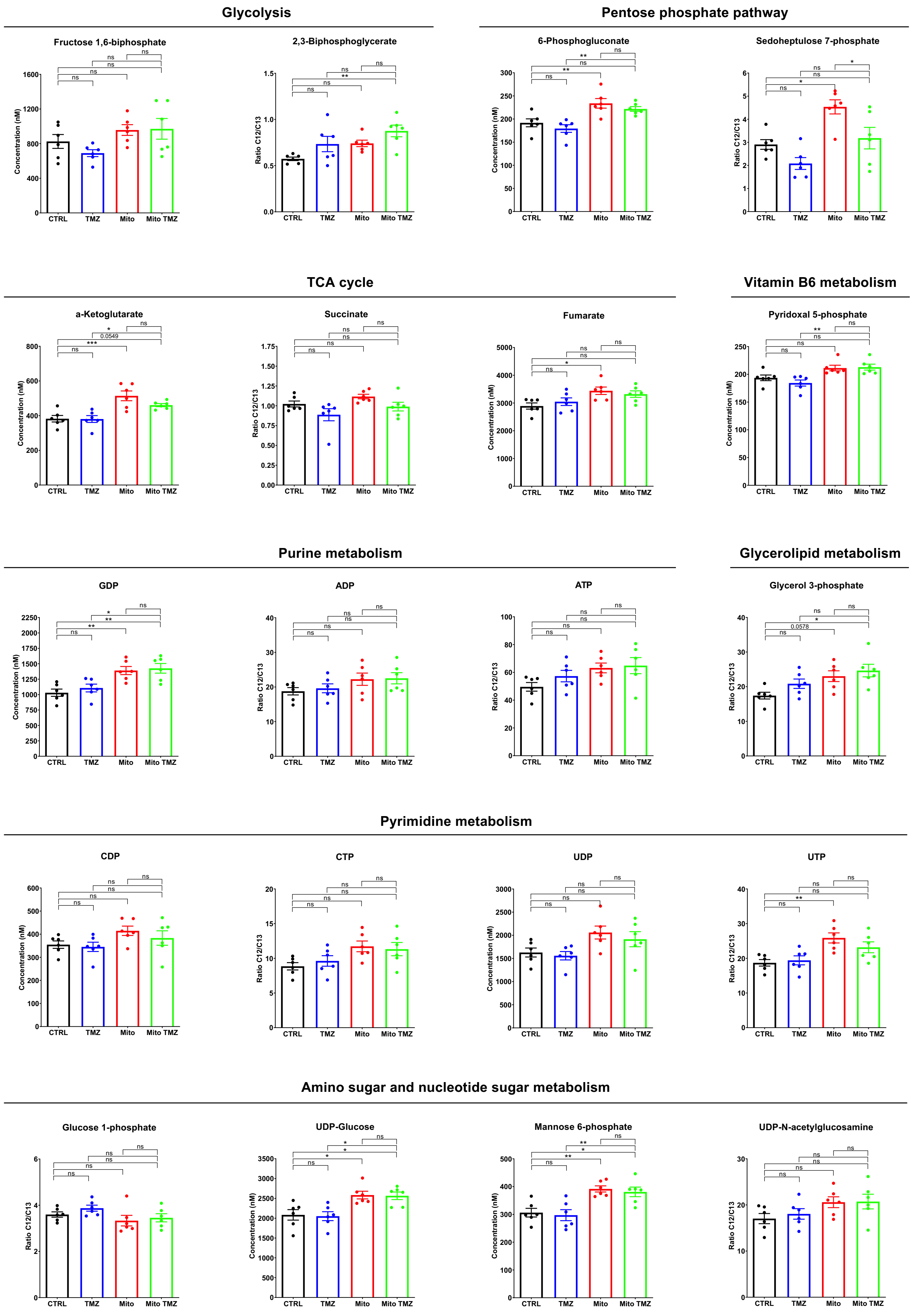

Metabolites produced by GSCs following MSC mitochondria acquisition and TMZ treatment
