# Supplementary material for: Mitochondria Transfer from Mesenchymal Stem Cells Confers Chemoresistance to Glioblastoma Stem Cells through Metabolic Rewiring
Source: Cancer Res Commun. 2023 Jun 14;3(6):1041–56. doi: 10.1158/2767-9764.CRC-23-0144 (PMC10266428; doi:10.1158/2767-9764.CRC-23-0144)
Supplement: Figure S5 — MSC mitochondria modify the usage of metabolites by GCSs in response to TMZ CTRL Mito TMZ CTRL Mito Mito TMZ (A) Metabolic substrate consumption of GSCs expressed as metabolite initial consumption rates (Biolog MitoPlates). Mean values ± SEM (4 independent experiments). (B) Production of cis-aconitate, succinate and L-malate expressed as a percentage of all TCA metabolites produced in each experimental condition. Tukey boxplots with one-way ANOVA, *p < 0.05, ***p < 0.001. [file crc-23-0144-s07.pdf]

Figure S5

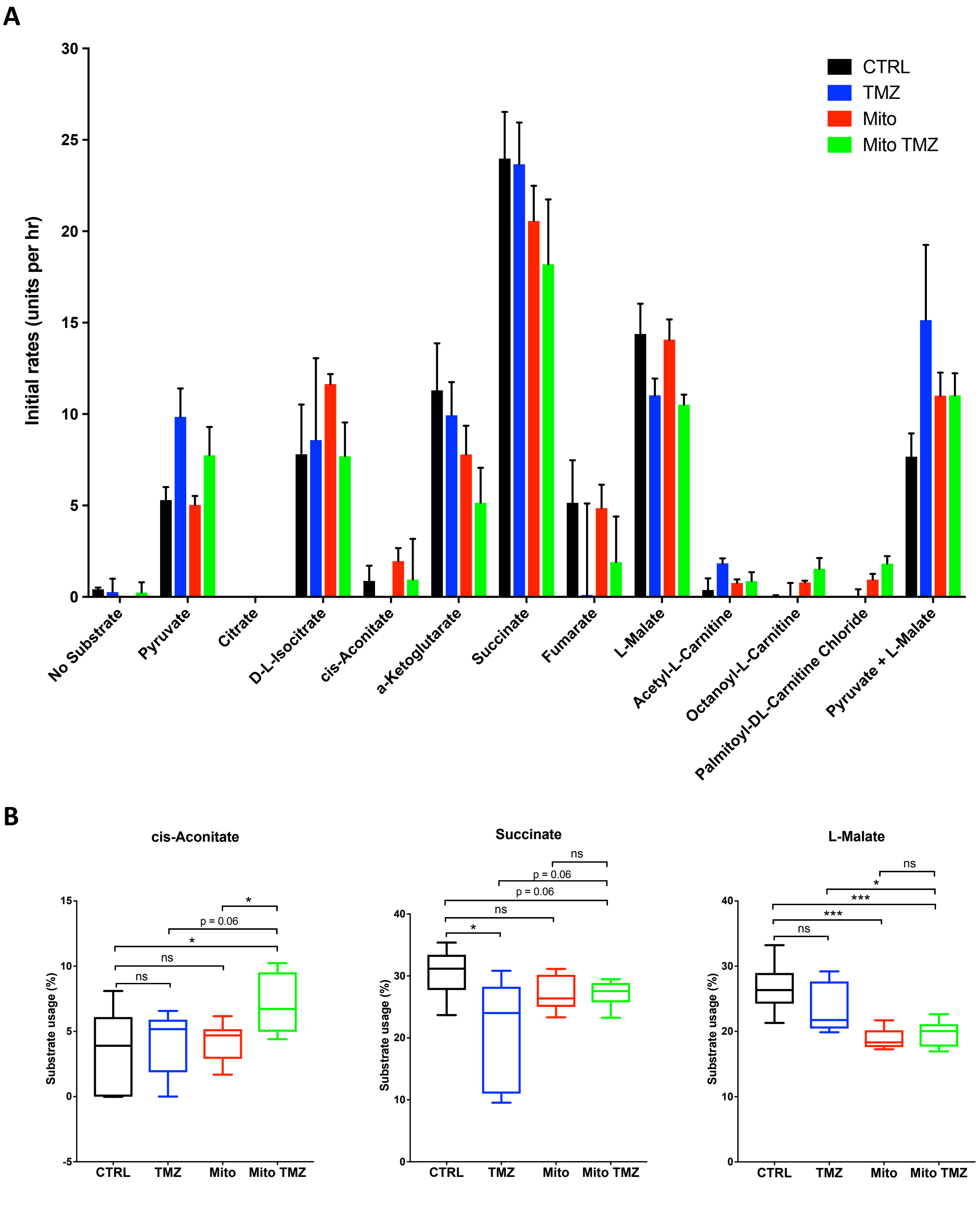

MSC mitochondria modify the usage of metabolites by GCSs in response to TMZ

(A) Metabolic substrate consumption of GSCs expressed as metabolite initial consumption rates (Biolog MitoPlates). Mean values ± SEM (4 independent experiments).

(B) Production of cis-aconitate, succinate and L-malate expressed as a percentage of all TCA metabolites produced in each experimental condition. Tukey boxplots with one-way ANOVA, *$p < 0.05$, ***$p < 0.001$.
