# Supplementary material for: Mitochondria Transfer from Mesenchymal Stem Cells Confers Chemoresistance to Glioblastoma Stem Cells through Metabolic Rewiring
Source: Cancer Res Commun. 2023 Jun 14;3(6):1041–56. doi: 10.1158/2767-9764.CRC-23-0144 (PMC10266428; doi:10.1158/2767-9764.CRC-23-0144)
Supplement: Figure S6 — Metabolite detection in resected GBM from 8 patients, at 1rst resection and at 2nd resection post-TMZ treatment. For each metabolite, the C12/C13 ratios normalized to tissue protein concentrations are indicated. [file crc-23-0144-s08.pdf]

Figure S6

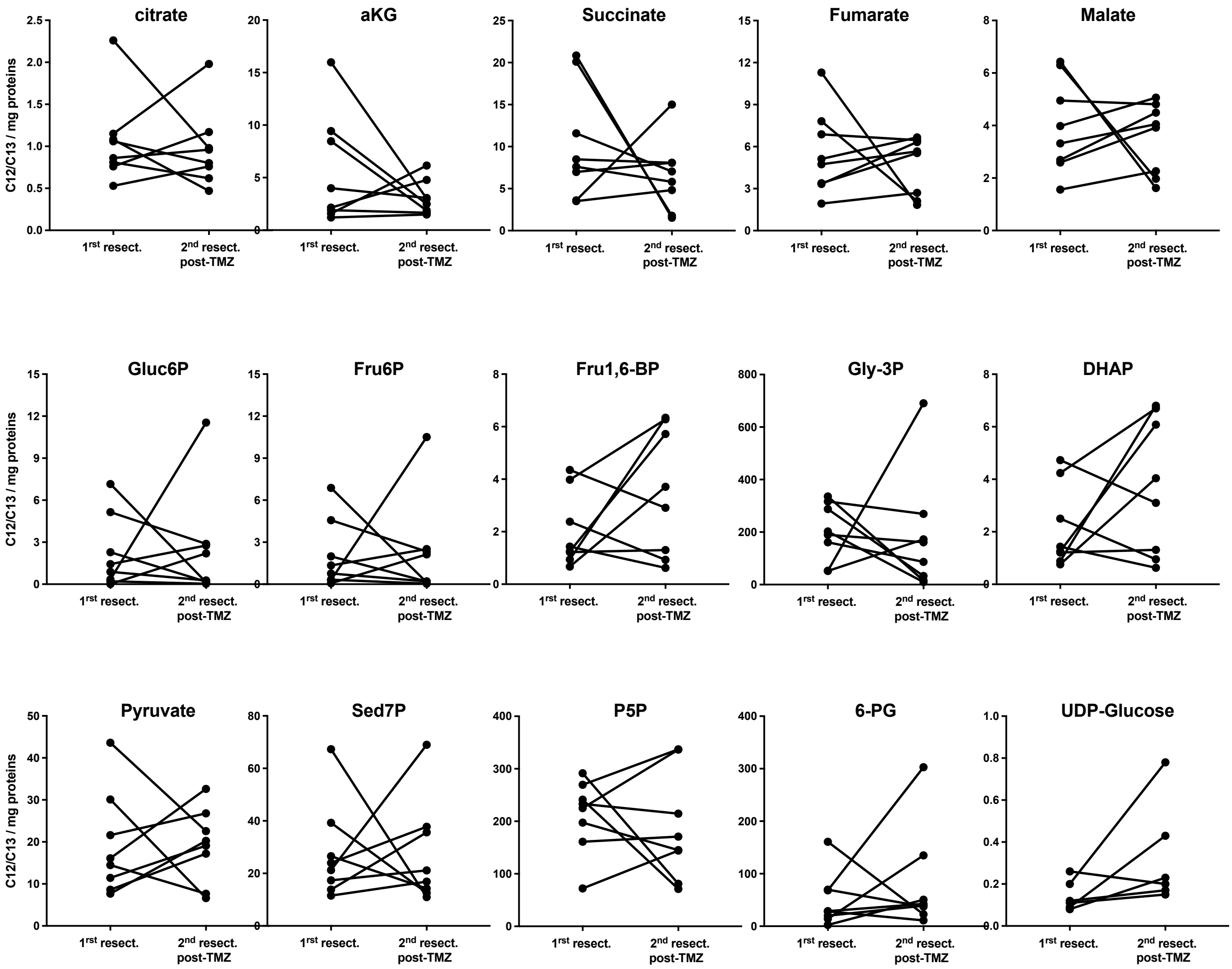

Metabolite detection in resected GBM from 8 patients, at 1rst resection and at 2nd resection post-TMZ treatment. For each metabolite, the C12/C13 ratios normalized to tissue protein concentrations are indicated.
